# REJENERA^©^, a multi-component bioflavonoid-based formula, alleviates osteoarthritis and provides chondroprotection by regulating the NLRP3-TXNIP-iNOS axis and inflammation: a comparative study with olive leaf nutraceuticals and ibuprofen

**DOI:** 10.1007/s10787-026-02314-2

**Published:** 2026-07-06

**Authors:** Huseyin Emre Tepedelenlioglu, Zubeyir Elmazoglu, Sanem Gulistan Saribas, Yildiz Tutuncu, Asli F. Ceylan, Çimen Karasu

**Affiliations:** 1 Department of Orthopedics and Traumatology, Ankara Dışkapı Training and Research Hospital, Ankara, Turkey; 2 Department of Pharmacology, Faculty of Pharmacy, Ankara Medipol University, Ankara, Turkey; 3 Department of Histology and Embryology, Faculty of Medicine, Health Sciences University Gulhane, Ankara, Turkey; 4 Department of Immunology, School of Medicine, Koç University Research Center for Translational Medicine (KUTTAM), Koç University, Istanbul, Turkey; 5 Department of Medical Pharmacology, Faculty of Medicine, Ankara Yıldırım Beyazıt University, Ankara, Turkey; 6 Department of Medical Pharmacology, Cellular Stress Response and Signal Transduction Research Laboratory, Faculty of Medicine, Gazi University, Ankara, Turkey; 7 Department of Medical Microbiology, Inonu University Faculty of Medicine, Malatya, Turkey

**Keywords:** Osteoarthritis, Inflammation, REJENERA, ZeyEX (olive leaf extract), *S*-allylcysteine, Palmitoylethanolamide, NLRP3, Ibuprofen

## Abstract

Osteoarthritis (OA) primarly involves the degradation of joint cartilage and requires new treatments. REJENERA^©^, a newly developed nutraceutical formula against OA, contains primarily olive leaf bioflavonoids ( ZeyEX^©^, quercetin and luteolin), *S*-allylcysteine, palmitoylethanolamide, l-proline, hyaluronic acid and boron. This study focuses on the efficacy of REJENERA in treating knee OA and aims to compare it with ZeyEX, NPROC^©^ (a product obtained by combining collagen-rich eggshell membrane with olive leaf extract), and IBUPROFEN in a rat OA model. OA was established by intra-articular injection of monosodium iodoacetate (MIA; 3  mg) into the right knee joints. Rats were either left untreated or treated orally for 12 weeks with REJENERA, ZeyEX, NPROC (300 mg/kg/day) or IBUPROFEN (3 mg/kg/day). MIA injection produced joint degeneration including increased fissure-index, osteophyte-score, and OARSI-score, joint swelling, synovial inflammation, proteoglycan loss, and decreased cartilage thickness. These histopathological abnormalities were partially but significantly alleviated by REJENERA and other treatments. Only ZeyEX significantly inhibited the OA-induced increase in IL-1β, IL-6, IL-10 and LPO in serum, and IL-6, TNF-α, and IFN-γ in synovial fluid. While increases in MMP-3 and MMP-9 were reduced with all treatments, MMP-13 was inhibited only by REJENERA. ZeyEX increased IL-2, NPROC increased IL-6, and IBUPROFEN inhibited IL-10. All treatments improved TIMP-1 levels; however, TXNIP was more significantly inhibited by ZeyEX, and NLRP3 by REJENERA. REJENERA’s anti-OA effects are accompanied by an increase in cartilage anabolic factors (Ki-67, type-II collagen, BMP-7) and inhibition of apoptosis. REJENERA offers a promising multi-targeted therapeutic approach to treating OA by blocking the iNOS-TXNIP-NLRP3 signaling axis, and reducing oxidative stress.

**Supplementary Information:**

The online version contains supplementary material available at https://doi.org/10.1007/s10787-026-02314-2.

## Introduction

Osteoarthritis (OA) is a chronic degenerative joint disease representing a significant health burden, leading to persistent pain and reduced mobility (Hunter and Bierma-Zeinstra 2019; Sharma 2021). Osteoarthritis (OA) is one of the most prevalent musculoskeletal disorders worldwide. Nevertheless, there is presently an absence of a disease-modifying pharmaceutical product in clinical practice (Skiöldebrand et al. 2023; Patel et al. 2026). Although OA pathogenesis is multifactorial, the progressive destruction and degradation of the cartilage extracellular matrix, triggered by factors such as inflammation, oxidative stress and metabolic disturbances, are considered central to disease onset and progression (Courties et al. 2015; Zhang et al. 2026). Mechanical overload, aging, obesity-related metabolic stress, low-grade inflammation, oxidative stress, and joint injury can impair chondrocyte homeostasis by altering key regulatory pathways, including NF-κB-mediated inflammatory signaling, MAPK activation, oxidative stress–responsive pathways, mitochondrial dysfunction, and inflammasome-related signaling. These changes promote chondrocyte catabolism, apoptosis, senescence, and extracellular matrix degradation, ultimately contributing to progressive cartilage degeneration and osteoarthritic joint damage (Lin et al. 2025).

Currently, the pharmacological treatment of OA mainly involves intra-articular injections of glucocorticoids, short-acting anti-inflammatory drugs, and hyaluronic acid-based supplements. However, these approaches do not prevent disease progression in the long term and are associated with adverse side effects, including cardiotoxicity, hepatotoxicity and gastrointestinal ulceration (Cai et al. 2022; Li et al. 2025; Boffa et al. 2025). Additionally, with the progression of OA, many traditional non-invasive strategies often fail to provide adequate symptomatic relief. In light of these limitations, there is growing interest in the use and investigation of phytochemical combinations, particularly due to their potential to exhibit synergistic interactions (Ashruf and Ansari 2022; Gan et al. 2025).

Recent studies on drug design have shown that phytochemical agents, especially in standardized and formulated pharmacognostic preparations, have advantages over synthetic drugs and biomaterial in terms of their pharmacodynamic effects and safety profiles (Liang et al. 2022; Bica et al. 2024). In fact, there is an increasing number of experimental and clinical studies, as well as practical applications showing that unique phytochemical formulations that regulate extracellular matrix metabolism, control the expression of inflammatory cytokines, maintain chondrocyte homeostasis by preventing early apoptosis, and stimulate chondrogenesis may be effective remedies compared to synthetic agents that interfere with only one pathway in the cellular mechanisms of OA and consist of only one active substance (Zhou et al. 2025; Gan et al. 2025; Patel et al. 2026). Polyphenolic phytochemicals, are well known for their potent anti-inflammatory and antioxidant activities, have recently been studied for their potential use in the treatment of degenerative musculoskeletal pathologies such as OA and osteoporosis (Wang et al. 2025). Some clinical studies have also been conducted using herbal remedies with a high phenolic content that are employed in Ayurvedic medicine (Rai et al. 2025). Polyphenolic flavonoids are recommended for long-term use in OA, in combination with anti-inflammatory and analgesic drugs already in clinical use or as an alternative to other products such as glucosamine and chondroitin, which are believed to improve the structure and flexibility of cartilage (Valsamidou et al. 2021). Recent advances in pharmacology and meta-analyses point to polyphenolic phytochemicals as promising therapeutic agents in terms of effectiveness and safety (Valsamidou et al. 2021; Naselli et al. 2023; Rahaman et al. 2025).

REJENERA is a novel patent-pending formula designed to treat degenerative joint diseases. REJENERA (supplied by Farmasens Co. Ltd. Turkey) contains primarly patented an olive (*Olea europaea* L.) leaf extract (ZeyEX^©^) standardized to oleuropein content (≥ 40% oleuropein). REJENERA also consist of ZeyEX bioflavonoids (luteolin and quercetin), *S*-allylcysteine, palmitoylethanolamide, l-proline, boron, and hyaluronic acid. We have already shown the anti-OA effects and action mechanisms of ZeyEX, its bioflavonoids (quercetin, luteolin, verbascoside and hydroxytyrosol), and *S*-allylcysteine in primary chondrocytes and synoviocytes cultures from knee joint cartilages of stage-4 OA patients (Goker et al. 2018; Elmazoglu et al. 2018, 2019, 2020a, b, 2021). ZeyEX targets multiple signaling pathways leading to cellular pathology in OA. *S*-allyl cysteine, a key component of REJENERA, regulates chondrocyte metabolism, inhibits oxidative stress, inflammation and apoptosis in chondrocytes (Elmazoglu et al. 2021; Shao et al. 2020).

The primary objective of this study was to evaluate the chondroprotective and anti-inflammatory effects of orally administered REJENERA in an MIA-induced rat model of knee OA . As a secondary objective, the effects of REJENERA were compared with those of ZeyEX, its eggshell membrane-encapsulated form NPROC, and ibuprofen using structural, histological, biochemical, and immunohistochemical outcome measures. In addition, selected mechanistic markers related to inflammation, matrix degradation, apoptosis, and the TXNIP/NLRP3/iNOS axis were examined to explore the biological pathways associated with the observed treatment effects.

## Materials and methods

### Animals and experimental groups

A total of 54 male Wistar rats (250–300 g upon arrival; GUDAM, Gazi University, Ankara) were housed in Plexiglass cages, three rats per cage, in climate-controlled colony rooms (22 ± 2 °C; 50% humidity), on a 12 h/12 h light/dark cycle (light phase: 07:00–19:00 h), with free access to tap water and food, 24 h/day throughout the study, with no fasting periods. The sample size was determined before the experiment based on the 3R principle and the minimum number of animals required to detect biologically meaningful differences among groups. Because the study included six experimental groups, sample-size estimation was based on one-way ANOVA for the primary structural outcomes, including macroscopic cartilage/bone damage, OARSI score, cartilage thickness, and inflammatory/catabolic biomarkers. Assuming a large expected effect size based on previous MIA-induced OA studies and our preliminary in vitro/in vivo observations, an effect size of f = 0.55, α = 0.05, and 80% statistical power required a minimum total sample size of approximately 48 animals. To compensate for possible biological variability, technical loss during histological processing, or unusable samples, 54 rats were included, with 9 rats per group. This sample size was considered sufficient for detecting major treatment-related effects while avoiding unnecessary animal use. Rats were fed a standard laboratory diet and all rats were acclimated for 7 days before starting the experiments. All animal procedures were conducted in accordance with the NIH guide for the Care and Use of Laboratory Animals (NIH Publications No. 8023, revised 1978). Randomization was performed by a researcher who was not involved in outcome assessment. The cages were color-coded for blinding purposes throughout the study. All experimental protocols were approved by the Gazi University Ethics Committee on the Use of Laboratory Animals (G.Ü.ET-21.081; Date: 23.12.2021). The rats were randomly divided into six experimental groups (n = 9 rats per group) using a computer-generated randomization sequence:

- Group 1 (*Sham*): Healthy rats without mono-iodoacetate (MIA) (Sigma-Aldrich, Germany) induction. 50 µL of phosphate-buffered saline solution (PBS) was injected once into the right knee. During the treatment procedures, 0.8 mL of PBS + 0.2% carboxymethyl cellulose (CMC) solution was administered p.o. for 12 weeks. This group served as the sham-operated reference group.
- Group 2 (*OA*): Osteoarthritis (OA) induced by MIA. A single intra-articular injection of MIA (3 mg, 50 μL in PBS) was injected into the right knee joint. Three days after the injection, 0.8 mL of PBS + CMC solution was given p.o. for 12 weeks.
- Group 3 (*ZeyEX*): A single dose of MIA (3 mg, 50 μL in PBS) was injected into the right knee joint. Three days after the injection, ZeyEX (300 mg/kg body weight/day) prepared in 0.8 mL PBS + CMC solution was administered p.o. for 12 weeks.
- Group 4 (*NPROC*): A single dose of MIA (3 mg, 50 μL in PBS) was injected into the right knee joint. Three days after the injection, NPROC (300 mg/kg body weight/day) prepared in 0.8 mL PBS + CMC solution was administered p.o. for 12 weeks.
- Group 5 (*REJENERA*): A single dose of MIA (3 mg, 50 μL in PBS) was injected into the right knee joint. Three days after the injection, REJENERA (300 mg/kg body weight/day) prepared in 0.8 mL PBS + CMC solution was administered p.o. for 12 weeks.
- Group 6 (*IBUPROFEN*): A single dose of MIA (3 mg, 50 μL in PBS) prepared in PBS was injected into the right knee joint. Three days after the injection, IBUPROFEN (3 mg/kg body weight/day) prepared in 0.8 ml PBS + CMC solution was administered p.o. for 12 weeks.

The 12-week duration was selected to evaluate treatment effects during the chronic structural phase of MIA-induced OA rather than the early inflammatory phase. Because MIA-induced pathology is dose- and time-dependent, longer follow-up allows assessment of progressive cartilage degeneration, subchondral bone changes, synovial alterations, and osteophyte formation (Udo et al. 2016; Yoh et al. 2022; Bryk et al. 2021). Therefore, this time point was considered appropriate for evaluating sustained structural, inflammatory, and catabolic changes and the potential chondroprotective effects of oral treatments. However, because MIA induces OA through chemically mediated chondrocyte injury, the model was interpreted as a chronic chemically induced OA-like model rather than a complete representation of slowly progressive human OA. The selected doses for ZeyEX, NPROC, and REJENERA were determined based on our previous findings from cell culture studies (Elmazoglu et al. 2019, 2020a, b, 2021) and previous in vitro findings from chondrocyte/synoviocyte models, available experimental studies using olive leaf polyphenols and related nutraceutical compounds, and feasibility of repeated oral administration in rats (Szychlinska et al. 2019; Stevens et al. 2021). Because direct conversion from cell-culture concentrations to animal dosing is not linear and is influenced by absorption, metabolism, tissue distribution, and formulation-dependent bioavailability, the selected dose of 300 mg/kg/day was used as a standardized oral dose to allow comparative evaluation of the three pharmacognostic preparations under the same experimental conditions. However, because these preparations differ in composition and active constituent content, the same nominal dose should not be interpreted as equivalent exposure to each active compound. The IBUPROFEN dose was selected using body surface area–based interspecies dose conversion. Human-equivalent dose was calculated according to the formula: HED (mg/kg) = animal dose (mg/kg) × [animal Km/human Km]. Using the standard Km values for rats and adult humans (rat Km = 6; human Km = 37), the rat dose of 3 mg/kg/day corresponds to an HED of approximately 0.49 mg/kg/day, or about 29–34 mg/day for a 60–70 kg adult. This dose was therefore selected as a low-dose positive-control anti-inflammatory comparator rather than as a full analgesic-equivalent human dose (Reagan-Shaw et al. 2008; Nair and Jacob 2016). IBUPROFEN was chosen as a positive control in this study because it is a nonsteroidal analgesic anti-inflammatory drug used in the treatment of OA. The selected ibuprofen dose (3 mg/kg) corresponds to the lowest effective dose that maintains anti-inflammatory activity in rats (Craft et al. 2021).

The agents used in the treatment were prepared freshly each day and given to the rats orally (per os) at the same time each day.

### Osteoarthritis induction

OA was induced in the right knee joint of rats in Groups 2–6. Rats were anesthetized using intraperitoneal injections of a combination containing 75 mg/kg of ketamine and 10 mg/kg of xylazine. The right knee area was shaved and aseptically prepared with 70% ethanol and povidone-iodine. A single intra-articular injection of 3 mg MIA (dissolved in 50 µL of PBS) was administered through the patellar ligament using a 26.5-gauge needle. The needle was inserted through the patellar ligament into the intra-articular space, and the solution was injected slowly. Successful intra-articular delivery was confirmed by minimal injection resistance and absence of subcutaneous bleb formation. An equivalent volume of PBS was injected into the right knees of control rats (Sham). After injection, the knee was gently flexed and extended for 30 s to distribute the MIA. Rats were monitored daily for signs of distress or infection. Given that the study was designed as a prevention model, treatment commenced three days after the MIA injection (Orhan et al. 2022).

### Treatment materials

ZeyEX, NPROC, and REJENERA were used as standardized pharmacognostic preparations supplied by their respective manufacturers. ZeyEX© is a dry extract of olive leaves (*Olea europaea* L.) standardized according to its oleuropein content. It is patented according to its production method (Patent No: TR 2013 15662 B) and was supplied by Farmasens Co Ltd, Ankara, Turkiye. According to the company, the main phenolic compounds of the ZeyEX used in this study include 60% oleuropein, 6% hydroxytyrosol, 0.8% luteolin, 14% luteolin 7-glucoside, 0.1% quercetin, 0.3% apigenin, 14% apigenin 7-glucoside, 1.5% isoacteoside, 1.4% catechin. Details regarding the biological activity and chemical composition of ZeyEX have previously been reported in various cellular degeneration models other than OA (Bali et al. 2014; Torul et al. 2020).

NPROC© consists of olive leaf dry extract combined with eggshell membrane, which is used as a collagen-rich carrier matrix (Patent Pending-Application No. TR 2021/002227, is a product developed by Bionley Co Ltd, Izmir, Turkey, using green nanotechnology). This formulation is designed to combine olive leaf polyphenols with structural components derived from eggshell membrane and potentially improve gastrointestinal stability and distribution. Characterization studies revealed that olive leaf polyphenols was successfully adsorbed onto the eggshell membrane, and cytotoxicity and antimicrobial studies showed that the prepared olive leaf-loaded membranes possessed bioactive properties (antioxidative and antimicrobial) (Bayraktar et al. 2021). The membrane was chosen due to its collagen and hyaluronic acid content, and this type of microencapsulation is performed to enhance the absorption of olive polyphenols from the gastrointestinal system (González et al. 2019). In the materials tested, the total phenol content was 59.90 for ZeyEX and 77.85 for NPROC [Gallic acid equivalent, (mg/g)] (All samples were measured at a concentration of 1 mg/mL. and dissolved in 100% methanol, and the measurements were taken at a wavelength of 725 nm.).

REJENERA© is a multi-component formulation developed for degenerative joint disorders and contains ZeyEX®, black garlic extract standardized for *S*-allylcysteine, quercetin, luteolin, palmitoylethanolamide, l-proline, hyaluronic acid, and boron. REJENERA is developed for use against OA (Patent Pending-Application No. TR 2020/009855) and supplied by Farmasens Co Ltd, Ankara, Turkiye. The final formulation of each REJENERA^©^ capsule contains ~ 15% ZeyEX, 8% black garlic extract (~ 0.1% *S*-allylcysteine), 8% quercetin, 5% l-proline, 5% hyaluronic acid, 3% luteolin, 3% boron and 20% palmitoylethanolamide. REJENERA is formulated in capsule form for use as a nutritional supplement in humans and therefore contains excipients. The contents of the capsule were used in rats treated with REJENERA.

### Assessment of joint oedema

To determine the oedema characteristic of inflammation following MIA injection into the right knee joints, the joints were measured using a calliper (Deqing LUFEN Electronic & Technology Co., Ltd.). For comparison, measurements were also taken on the left knee joints. The caliper tips were positioned based on the outer borders (periphery) of the medial and lateral condyle, and then the measurements were taken. Measurements were performed on the same day and at the same time each week throughout the treatment period. Joint diameter ratios were calculated according to Logashina et al. and plotted as weekly changes: MIA-injected joint diameter/intact joint diameter × 100 (Logashina et al. 2021).

### Serum, synovial fluid and knee isolation and processing

At the end of the 12-week treatment period, rats were deeply anesthetized with an intraperitoneal injection of ketamine/xylazine mixture (75 mg/kg ketamine and 10 mg/kg xylazine). Adequate depth of anesthesia was confirmed by the absence of pedal withdrawal reflex before sample collection. Blood samples were obtained as a terminal procedure by intracardiac puncture from the cardiac chamber using a sterile syringe and needle while the animals were under deep anesthesia. Following blood collection, animals were euthanized by exsanguination under anesthesia. The collected blood was transferred into serum tubes and allowed to clot at room temperature. Serum was then separated by centrifugation and stored at − 80 °C until biochemical analysis. After blood collection, the right knee joint was punctured under aseptic conditions using a 27-gauge needle. Synovial lavage was performed by injecting 200 μL of phosphate-buffered saline (PBS) containing 1.5% potassium ethylenediaminetetraacetic acid (K_2_EDTA) into the joint cavity. The joint was gently flexed and extended to facilitate mixing of the lavage fluid with synovial fluid, and the fluid was then aspirated. This lavage–aspiration procedure was repeated twice, as previously described by Lakshmanan et al. (2020). Synovial fluid aspirates were centrifuged at 3000 rpm at 24 °C for 15 min, and the supernatants were collected and stored at − 80 °C until analysis. After serum and synovial fluid collection, knee joints were isolated for radiographic, macroscopic, histological, immunohistochemical, and molecular analyses.

The right and left knees were photographed from various angles. Following these procedures, X-rays of the joints were taken using the GC70 Digital Radiography system (Samsung). Then; macroscopic assesment of the joints were performed via macroscopic cartilage and bone scoring system (Udo et al. 2016). Afterward, some joints were stored on shelves at − 80 °C to obtain cartilage. Furthermore, the medial condyle of the femur and tibia from five animals was separated for histomorphological and immunohistochemical analyses, and bone-cartilage tissue from four animals was reserved for Western blot experiments.

### Biochemical analysis in serum samples

Serum levels of lipid peroxidation products (LPO), matrix metalloproteinases (MMP-3, MMP-9, and MMP-13), tissue inhibitor of metalloproteinase-1 (TIMP-1), and inflammatory cytokines (IL-1β, IL-6, and IL-10) were measured using commercially available rat-specific assay kits according to the manufacturer’s instructions. The following kits were used: rat MMP-3 ELISA kit (E-EL-R0619; detection range, 0.16–10 ng/mL; sensitivity, 0.10 ng/mL), rat MMP-9 ELISA kit (E-EL-R3021; detection range, 7.81–500 ng/mL; sensitivity, 4.69 ng/mL), rat MMP-13 ELISA kit (E-EL-R0045; detection range, 0.31–20 ng/mL; sensitivity, 0.19 ng/mL), rat TIMP-1 ELISA kit (E-EL-R0540; detection range, 0.16–10 ng/mL; sensitivity, 0.10 ng/mL), rat IL-1β ELISA kit (E-EL-R0012; detection range, 31.25–2000 pg/mL; sensitivity, 18.75 pg/mL), rat IL-6 ELISA kit (E-EL-R0015; detection range, 12.5–800 pg/mL; sensitivity, 7.5 pg/mL), rat IL-10 ELISA kit (E-EL-R0016; detection range, 31.25–2000 pg/mL; sensitivity, 18.75 pg/mL), and LPO colorimetric assay kit (E-BC-K176-M; detection range, 0.70–80 µmol/L; sensitivity, 0.70 µmol/L) (Elabscience Biotechnology Inc., Houston, TX, USA). For ELISA measurements, serum samples and standards were brought to room temperature before analysis. Unless otherwise required by the kit protocol, serum samples were analyzed without pre-assay dilution; samples exceeding the upper limit of the standard curve were diluted with the appropriate sample diluent and recalculated using the corresponding dilution factor. Briefly, 100 µL of standards or serum samples were added to antibody-precoated 96-well plates and incubated at 37 °C according to the manufacturer’s protocol. After washing, biotinylated detection antibody and avidin–horseradish peroxidase conjugate were added sequentially. Following the final wash step, substrate solution was added, the reaction was stopped with stop solution, and absorbance was measured at 450 nm using a BioTek ELX800 Absorbance Microplate Reader (BioTek Instruments Inc., Winooski, VT, USA). LPO levels were determined using the colorimetric assay protocol and absorbance was measured at 580–590 nm. All samples, standards, and blanks were assayed in duplicate wells on the same plate for each analyte. The mean value of the duplicate wells was used for statistical analysis. Concentrations were calculated from standard curves generated using the kit standards. Cytokine levels were expressed as pg/mL, MMP-3, MMP-9, MMP-13, and TIMP-1 levels as ng/mL, and LPO levels as µmol/L. For normalization, total protein concentration in each serum sample was determined using the protein assay recommended in the corresponding kit protocol, and results were normalized to total protein content when applicable.

### Evaluation of inflammatory parameters in synovial fluid samples

The simultaneous quantification of IFN-γ, IL-1β, IL-6, TNF-α, IL-10 and IL-2 in synovial fluid samples was performed using the Rat Luminex Discovery Assay (catalog no. LXSARM; R&D Systems, Minneapolis, MN, USA) according to the manufacturer’s instructions. Synovial fluid lavage supernatants were thawed and centrifuged briefly to remove particulate material before analysis. Samples were diluted with Calibrator Diluent RD6-52 according to the manufacturer’s recommendations. Assay sensitivities and detection ranges for each analyte were those specified in the lot-specific Certificate of Analysis supplied with the kit (lot no. L156499). Following incubation and washing steps, samples were analyzed within 90 min using a Luminex 100 analyzer (Luminex Multiplex Assay System; Luminex Inc.). Six biological samples from each experimental group were analyzed, and all standards, blanks, and samples were assayed in duplicate wells. Median fluorescence intensity (MFI) values were processed using Quantist Luminex Analysis software, which performed background correction and generated analyte-specific standard curves using a five-parameter logistic (5-PL) model. Cytokine concentrations were calculated from these curves and expressed as pg/mL. For each sample, the final concentration used for statistical analysis represented the mean value obtained from duplicate measurements. When both duplicate measurements yielded values below the lower limit of detection, the corresponding cytokine concentration was recorded as zero for statistical analysis.

### Western blot

Ground cartilage tissue was homogenized on ice in lysis buffer containing 50 mM Tris–HCl, 150 mM NaCl, 1 mM EDTA, 1% Triton X-100, and 0.1% SDS at pH 7.4, supplemented with protease and phosphatase inhibitor cocktails. The lysates were centrifuged to remove insoluble debris, and total protein concentrations were determined using a BCA protein assay kit according to the manufacturer’s instructions (Thermo Fisher Scientific, USA). Equal amounts of total protein (20 μg per lane) were mixed with loading buffer, denatured, and separated by SDS–polyacrylamide gel electrophoresis. A 10% gel was used for NLRP3 detection, whereas 12% gels were used for TXNIP and β-actin detection. Proteins were then transferred onto nitrocellulose membranes (Sartorius Stedim, Göttingen, Germany) using a wet-transfer system.

To avoid signal interference caused by the use of rabbit-derived primary antibodies for all targets, NLRP3, TXNIP, and β-actin were detected on separate membranes or on membrane sections cut according to the expected molecular weight of each protein before antibody incubation. Each membrane or membrane section was incubated with only one primary antibody. After transfer, membranes were blocked for 1 h at room temperature in TBS-T containing 5% skim milk and then incubated overnight at 4 °C with the following primary antibodies: anti-NLRP3 rabbit polyclonal antibody (1:1000, bs-10021R, Bioss Inc.), anti-TXNIP rabbit polyclonal antibody (1:1000, bs-3897R, Bioss Inc.), and anti-β-actin rabbit monoclonal antibody (1:1000, A026, ABclonal Technology). After washing three times with TBS-T, membranes were incubated for 2 h at room temperature with horseradish peroxidase-conjugated goat anti-rabbit IgG (H + L) secondary antibody (AS014, ABclonal Technology) diluted in blocking buffer according to the manufacturer’s recommendation. Following secondary antibody incubation, membranes were washed three times for 5 min with TBS-T. Protein bands were visualized using an enhanced chemiluminescence detection substrate and imaged with a Gen-Box imagER TM Fx Imaging and Documentation System (Thermo Fisher Scientific Inc.). Densitometric analysis of band intensities was performed using ImageJ software. The intensity of each target protein band was normalized to the corresponding β-actin signal from the same sample, and the results were expressed as relative fold change compared with the sham control group. Following secondary antibody incubation, membranes were washed three times for 5 min with TBS-T. Protein bands were visualized using an enhanced chemiluminescence detection substrate and imaged with a Gen-Box imagER TM Fx Imaging and Documentation System (Thermo Fisher Scientific Inc.). Densitometric analysis of band intensities was performed using ImageJ software. The intensity of each target protein band was normalized to the corresponding β-actin signal from the same sample, and the results were expressed as relative fold change compared with the sham group.

### Histochemical studies

#### Hematoxylin–eosin (H&E) staining: determination of fissure index, osteophyte score, and synovial inflammation score

After blood and synovial fluid were collected, knee samples (5 animals from each group) were subjected to fixation and then decalcified with 8% formic acid.After approximately 40–45 days, the decalcified joints were processed using standard procedures and paraffin blocks were created. When the anterior and posterior horns of the medial meniscus began to separate, 4 µm thick sections were taken. Serial sections from paraffin blocks were used in histochemical studies. Hematoxylin–Eosin (H&E) was used to evaluate histomorphological changes, fissure index, osteophyte scoring, and synovial inflammation. Fissures or surface undulations in the upper region of the articular cartilage are also typical features of cartilage degeneration in OA. Using H&E staining, fissures were evaluated in terms of depth, branching, and vertical extension. To quantitatively describe the degree of superficial cracking, the fissure index (FI) was calculated as follows: FI = length of cartilage surface (L)/width of the measured area (W) (Pastoureau et al. 2010).

Osteophyte scoring was performed as a reflection of the thickness of inflammatory cells. Osteophyte scores in knee joint sections were evaluated according to previous studies (Grote et al. 2023; Glasson et al. 2010).The synovial inflammation score is a quantifiable metric of the severity of synovitis, or synovial inflammation, by evaluating the thickness and density of inflammatory cells and lining hyperplasia. High inflammation intensity accelerates damage to adjacent articular cartilage by releasing catabolic enzymes that degrade the cartilage matrix, fueling a progressive cycle of joint destruction (Sanchez-Lopez et al. 2022). The intensity of synovial inflammation was determined in articular cartilage sections according to a previously described method (Glasson et al. 2010; Grote et al. 2023).

#### Safranin-O/fast-green staining: determination of OARSI (Osteoarthritis Research Society International) scoring, cartilage thickness and proteoglycan loss

Safranin-O/fast green staining was used to assess cartilage thickness, proteoglycan loss, and OARSI (Osteoarthritis Research Society International) scoring, and to differentiate between cartilage and subchondral bone (Pritzker et al. 2006). Four-micron-thick sections taken from paraffin blocks were kept in Weigert’s iron hematoxylin solution for 10 min. After washing, the sections were kept in fast green solution for 5 min. After rinsing quickly with 1% acetic acid solution for no more than 10–15 s, they were kept in 0.1% safranin O solution for 5 min. After passing through an increasing alcohol series, the sections were brightened with xylene and covered with Entellan. The the degenerative changes in the medial femoral condyle, medial tibial plateau, lateral femoral condyle and lateral tibial plateau of the stained knee samples were scored according to the OARSI criteria (Table 1) and the groups were compared. Four representative sections, each 4 µm thick / 50 μm interval, were selected from each sample for quantitative analysis. Images of the knee joint obtained by Safranin O-Fast green staining were scored according to OARSI criteria. For histological illustration, representative sections were selected from animals exhibiting median histological scores within each group. Selection was performed after completion of blinded histological assessment to minimize selection bias.

**Table 1:** OA cartilage histopathology OARSI grade assessment methodology

Grade (basic feature)	Relevant criteria (tissue reaction)
0 (Normal)	Matrix normal, surface intact
1 (Superficial fibrillation)	Superficial fibrillation (wearing) in matrix, focal superficial matrix condensation
	Cells clumped, hypertrophic, apoptotic
2 (Deep fibrillation)	In addition to Grade 1:
	Deep fibrillation in superficial area
	Decreased Safranin O uptake in upper 1/3 of cartilage
3 (Deep clefts)	In addition to Grade 2:
	Vertical clefts in matrix towards middle area, branched clefts
	Decreased Safranin O uptake in upper 2/3 of cartilage
	Cartilage areas adjacent to clefts
4 (Erosion)	Loss of cartilage matrix, delamination of superficial layer, cyst formation in middle layer
5 (Sloughing)	Completely eroded unmineralized hyaline cartilage. The joint surface is mineralized cartilage or bone. Microfracture with repair limited to the bone surface
6 (Deformation)	Microfracture, repair and bone remodeling. Microfracture with fibrocartilaginous and bone repair extending over the previous surface

On the other hand, Safranin O-Fast green stain also highlights the proteoglycan content, consisting of sulfated glycosaminoglycan chains containing chondroitin sulfate and keratan sulfate. Fast green is used as a contrast marker for bone. Due to its high proteoglycan content, cartilage stains dark red with Safranin O, indicating active chondrocyte function. However, staining in cartilage decreases due to the reduced proteoglycan content of the cartilage matrix. In addition, calcified cartilage stains bright pink. Histopathological data were used to analyse the femoral condyles and tibial plateaus of the knee joint in vivo. Cartilage thickness serves as a primary metric for determining the severity of joint structural damage. Histologically, it is measured across the uncalcified and calcified cartilage zones using high-magnification microscopy. Here, cartilage thickness was measured in order to provide the clearest possible demonstration of cartilage erosion (Pritzker et al. 2006). The cartilage thickness was measured by taking four measurements from each sample. Proteoglycan loss was scored using Safranin O-Fast green staining images (0: normal, 1: minimal loss, 2: mild loss, 3: moderate loss, and 4: severe loss) (Pritzker et al. 2006; Pinamont et al. 2020).

### Immunohistochemistry study and TUNEL assays

For immunohistochemical evaluations, 4 µm-thick polylysine-coated sections were deparaffinized, rehydrated, and subjected to heat-induced epitope retrieval in a microwave using a citrate buffer solution (pH 6.0). Endogenous peroxidase activity was quenched with 3% hydrogen peroxide. Sections were then incubated overnight at 4 °C with primary antibodies against collagen II (Col-2; Bs-0709R, Bioss), Ki-67 (Bs-2310R, Bioss), BMP7 (Bs-2242R, Bioss), and iNOS (Bs-2072R, Bioss) at a 1:200 dilution. Immune complexes were detected using a biotinylated secondary antibody (Cat. No. TP-125-BN, Lot No. PBN100121, Lab Vision, Thermo Scientific) followed by a streptavidin-peroxidase enzyme complex (Cat. No. TS-125-HR, Lot No. SHR100406, Lab Vision, Thermo Scientific), and visualized using 3,3′-Diaminobenzidine (DAB) chromogen. Apoptotic cells in rat articular cartilage were identified using the Apo-BrdU-IHC In Situ DNA Fragmentation Assay Kit (Cat. No. K403, BioVision) on 4 µm-thick sections, following the manufacturer’s instructions. For both assays, sections were counterstained with Mayer’s hematoxylin, dehydrated through a graded alcohol series, cleared in xylene, and mounted with Entellan. To ensure staining specificity, negative controls were processed simultaneously by omitting the primary antibodies or TdT enzyme, while previously validated positive tissue sections were included for each marker.

Digital images were captured using a computer-assisted light microscopy system under identical illumination, magnification, and exposure settings. Six distinct sections of the knee joint were evaluated per subject, and four random fields were analyzed per section. Quantitative analysis of Col-2, BMP7, and iNOS immunoreactivity was performed using ImageJ software (NIH, Bethesda, MD, USA). DAB-positive areas were quantified as the percentage of positive staining area within predefined regions of interest (ROIs) utilizing the Color Threshold function. Threshold parameters were optimized using negative control sections to eliminate non-specific background staining and remained constant throughout the analysis. Ki67 and TUNEL-positive cells were quantified and expressed as a percentage of total cells. All morphometric and quantitative analyses were performed by an investigator blinded to the experimental group allocations.

### Statistical analysis

General data processing and statistical analyses were performed using GraphPad Prism 9 software (GraphPad Inc., USA). A one-way analysis of variance (ANOVA) was used to compare parametric data that were normally distributed among groups. The means ± standard deviations were used for statistical analysis. Statistical significance was determined using a *p* value of less than 0.05. Statistical analyses and calculations for histochemical data were performed using IBM SPSS Statistics version 21 (IBM Corp., Armonk, NY, USA). Normality was evaluated using graphical analysis and the Shapiro–Wilk test. Continuous variables were described using mean ± standard deviation, median, minimum, and maximum values. Since the data displayed a non-normal distribution, the non-parametric Kruskal–Wallis test was used for multiple-group comparisons of both continuous variables and semi-quantitative ordinal categorical variables (OARSI score, proteoglycan loss, synovial inflammation score, and osteophyte score). When statistically significant differences were identified among the groups, post hoc pairwise comparisons were conducted using the Mann–Whitney U test with Bonferroni correction to identify the distinct groups. Ordinal variables were also summarized using frequencies, percentages, and median (minimum–maximum) values. Statistical significance was set at *p* < 0.05.

## Results

### Macroscopic morphologic changes in joints

**Fig. 1:**
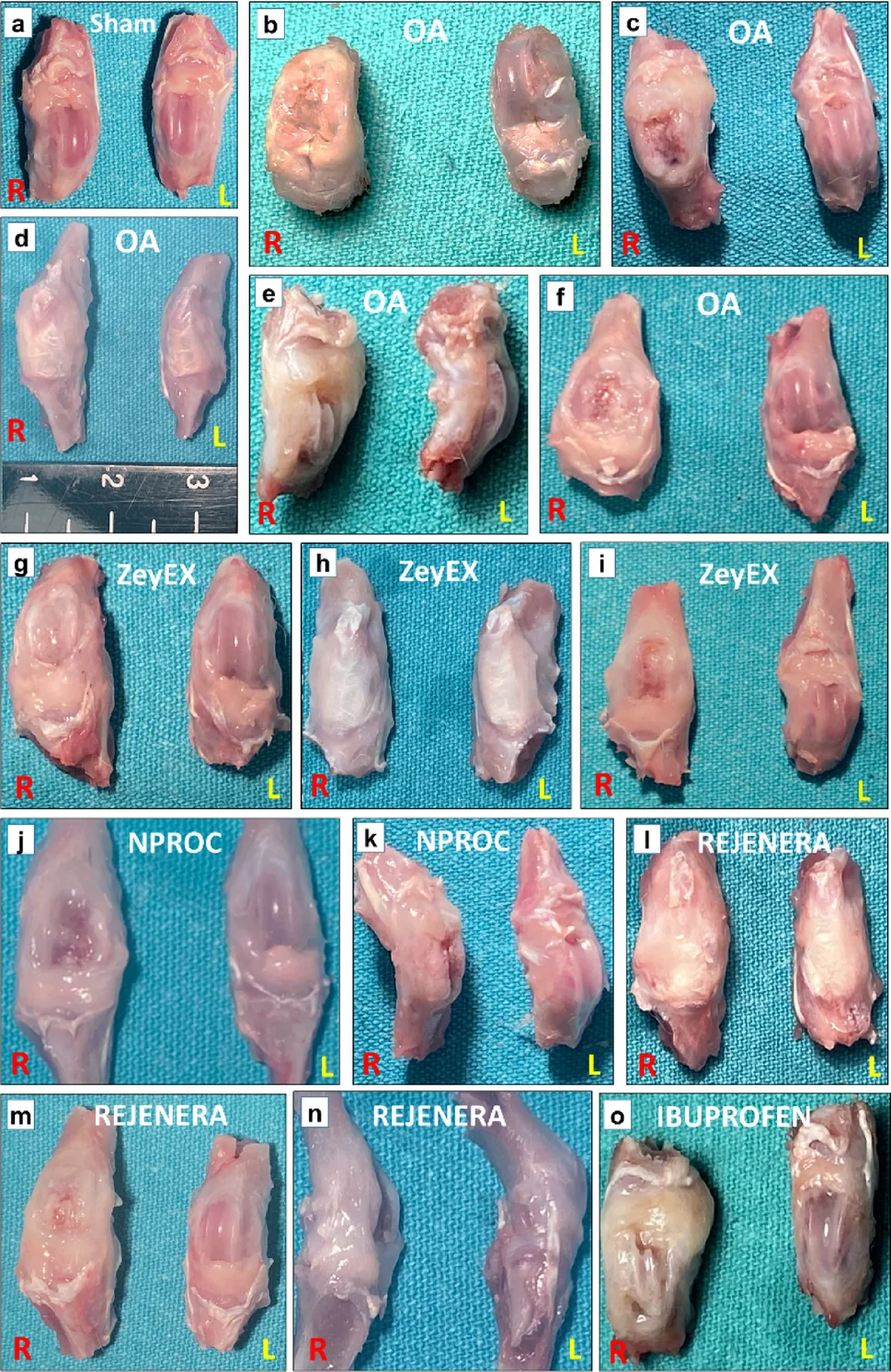
Effect of the treatments on the macroscopic view of articular cartilage damage in each group. Macroscopic examination of rat knee joints after 12 weeks of treatment showed that the right knee joints injected with MIA, compared to the left knees of the same animal and the right knees of sham rats, showed damage characterized by synovitis, cartilage loss, and pitting on the bone surfaces in the lateral medial condyles. Images reflecting synovial hypertrophy and chondromalacia in the femoral condyles of the right knee joints with OA were also present. Overall, the OA groups had a rough articular surface and osteochondral defect in the intercondylar space of the distal femoral condyle, and osteophyte formation was observed. Groups: Sham (**a**), non-treated osteoarthritis (OA, **b**–**f**), ZeyEX-treated OA (ZeyEX, **g**–**i**), NPROC-treated OA (NPROC, **j**, **k**), REJENERA-treated OA (REJENERA, **l**–**n**) and ibuprofen-treated OA (IBUPROFEN, **o**). In each photograph, the right knee joint (R) injected with MIA and the left knee joint (L) without MIA injection are shown side by side on the same animal

Following the 12-week period of treatment, macroscopic joint damage was semi-quantitatively evaluated using the Udo et al. macroscopic cartilage and bone scoring system (Udo et al. 2016). The sham group showed intact articular surfaces and received a score of 0, whereas untreated OA joints showed marked macroscopic degeneration, including cartilage erosion, surface irregularity, pitting, osteophyte-like changes, and occasional bone destruction, corresponding to scores of 3–5. Representative scores were lower in the treated groups, ranging from 2–3 for ZeyEX, 3–4 for NPROC, 1–2 for REJENERA, and 2–3 for IBUPROFEN. Overall, macroscopic damage scores differed significantly among groups by Kruskal–Wallis analysis (*p* < 0.001), and the untreated OA group had significantly higher scores than the sham group (*p* < 0.001). Compared with untreated OA, significant improvement was observed in the REJENERA and IBUPROFEN groups (*p* = 0.006 and *p* = 0.018, respectively), whereas the reductions in the ZeyEX and NPROC groups did not reach statistical significance (*p* = 0.083 and *p* = 0.221, respectively). No direct superiority among active treatments was inferred, as post hoc comparisons between treatment groups were not statistically significant (Fig. 1).

### X-ray imaging findings

**Fig. 2:**
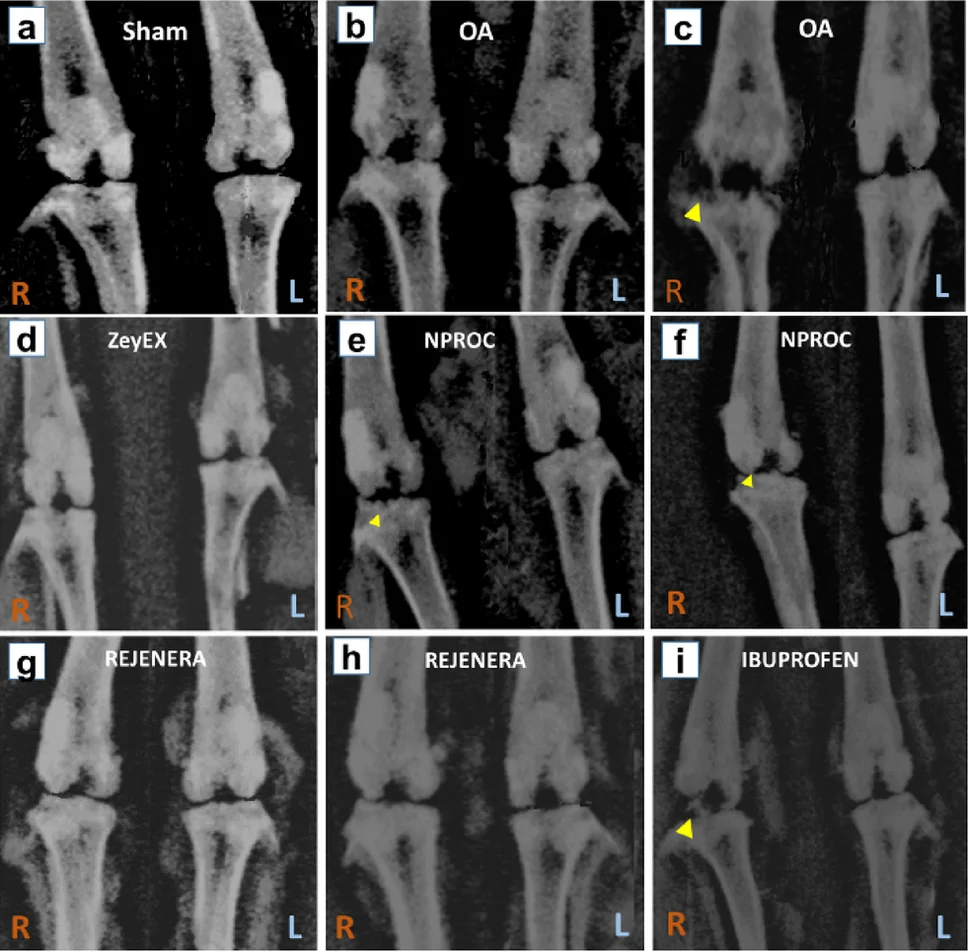
Representative radiographic images of the rats’ knee joints. The right knees of untreated OA rats showed narrow joint space, sclerosis, osteophyte formation, femoral condyle bone change, subchondral cyst formation in the medial tibia, and irregular cartilage surface with undefined contours compared to sham rats (yellow arrow heads). Groups: Sham (**a**), non-treated osteoarthritis (OA, **b**, **c**), ZeyEX-treated OA (ZeyEX, **d**), NPROC-treated OA (NPROC, **e**, **f**), REJENERA-treated OA (REJENERA, **g**, **h**) and ibuprofen-treated OA (IBUPROFEN, **i**)

After euthanizing the animals at the end of the 12th week, the knee joints were resected and radiographs were taken of the right (R) and left (L) knees side by side (Fig. 2). In untreated OA rats, compared to the sham rats, narrowing of the joint space, subchondral sclerosis and osteophyte formation, femoral condyle bone alteration, and irregular cartilage surface with undefined contours were observed. Treatments partially reduced osteophyte and sclerosis formation. In the treatment groups, morphological changes were observed to be milder (Fig. 2).

### Knee joint oedema and anthropometric assessment of inflammation

**Fig. 3:**
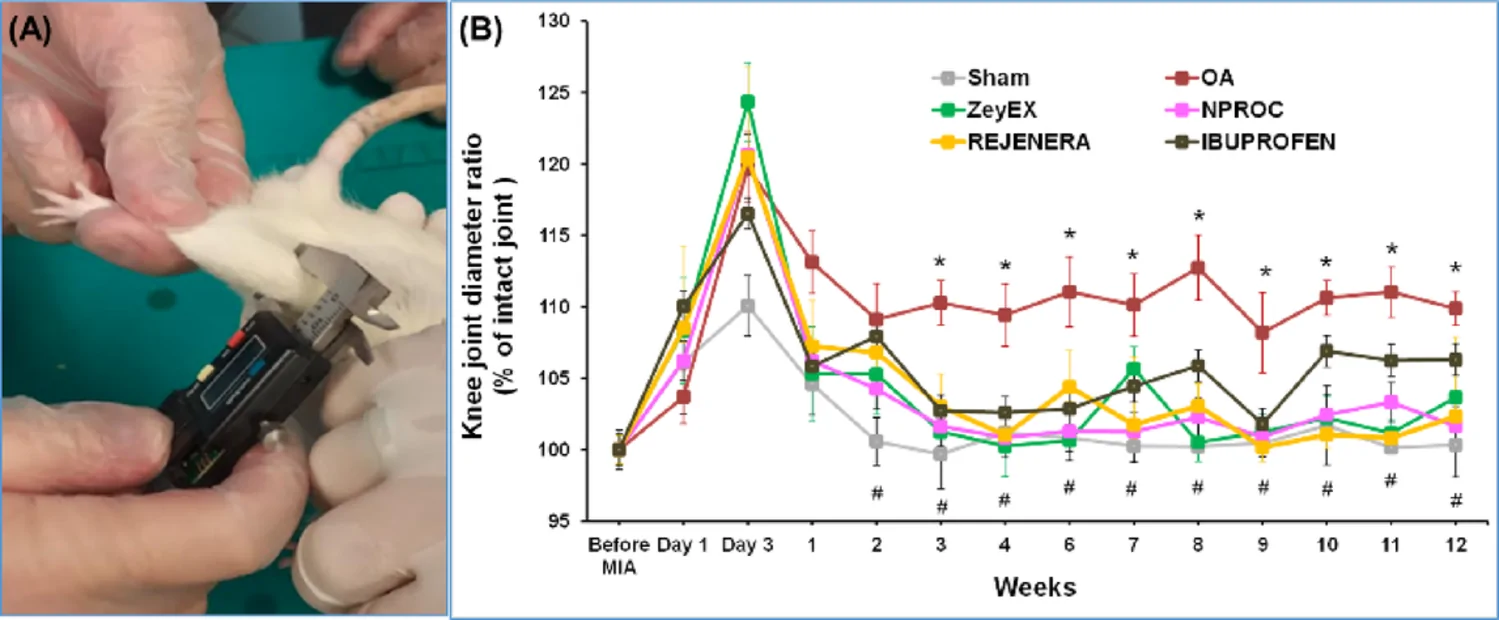
Joint diameter measurement methodology and results of quantitative analysis. (**A**) Representative illustration of measuring joint diameter using a caliper. (**B**) Group means of weekly changes calculated in right and left joint diameter ratios, [(MIA injected joint diameter/intact joint diameter) × 100]. n = 9 for each experimental group. Data are expressed as mean ± SD. *p < 0.05 *vs*. Sham; *p < 0.05 *vs*. OA (ANOVA)

The diameters (in mm) of the MIA-injected (right) and intact (left) joints were measured with a caliper before injection, on days 1 and 3 after MIA injection, and weekly thereafter (Fig. 3A). Inflammation was assessed by measuring the increase in the diameter of the injected joint and calculating the ratio of this to the diameter of the intact joint (as a percentage of the latter). The changes in joint diameter ratios are shown in Fig. 3B. The findings revealed that the weekly average right joint diameter ratios in the untreated OA group differed from those in the treated OA groups at least at week 3 and in subsequent weeks. Although there was no statistically significant difference when comparing the mean weekly changes in right joint diameter ratios between the treatment groups, overall, the mean weekly increase in right joint diameter ratios at the end of treatment was lower in each treatment group compared to the OA group (*p* < 0.05) (Fig. 3B).

### Effects of ZeyEX, NPROC, REJENERA and IBUPROFEN on inflammatory and injury-linked parameters in serum

**Fig. 4:**
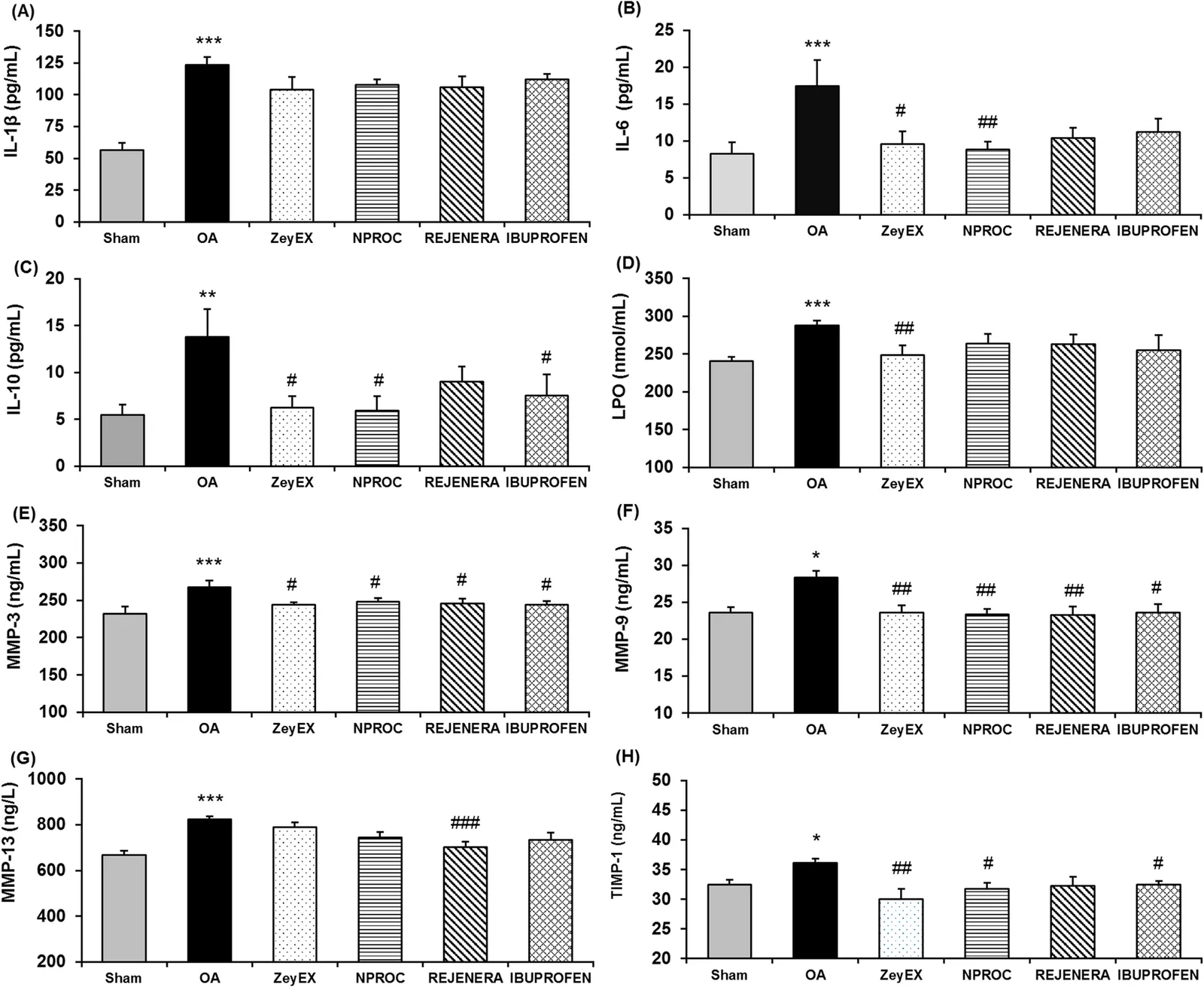
Changes in serum levels of  (**A**) IL-1β, (**B**) IL-6, (**C**) IL-10, (**D**) LPO, (**E**) MMP-3, (**F**) MMP-9, (**G**) MMP-13 and (**H**) TIMP-1 in Sham , osteoarthritis (OA), ZeyEX, NPROC, REJENERA and IBUPROFEN treatment groups. n = 9 for each experimental group. Data are expressed as mean ± SD.*p < 0.05, **p < 0.01, ***p < 0.001 *vs*. Sham; ^#^*p* < 0.05, ^##^*p* < 0.01, ^###^*p* < 0.001 *vs*. OA (ANOVA, Tukey’s post-hoc test)

As demonstrated in Fig. 4A, IL-1β levels were found to be significantly elevated in the OA group in comparison with the sham group (*p* < 0.001). The present study demonstrates that ZeyEX treatment was the only intervention capable of statistically significantly preventing the increase in IL-1β levels (*p* < 0.05 *vs*. OA). The serum levels of pro-inflammatory cytokine IL-6 were significantly increased in OA group (*p* < 0.001) (Fig. 4B). The increase in IL-6 levels induced by OA was statistically significantly reduced by ZeyEX (*p* < 0.05) and NPROC (*p* < 0.01) treatments. IL-10 levels were significantly increased in OA group (*p* < 0.01) (Fig. 4C). In the groups treated with ZeyEX, NPROC and IBUPROFEN, a significant decrease in IL-10 levels was observed in comparison to the untreated OA group (*p* < 0.05), Furthermore, increased serum levels of lipid peroxidation products (LPO) were detected in the OA group in comparison to the sham group (*p* < 0.001) (Fig. 4D). Despite the tendency for an increase in LPO levels to decrease with all treatments, it was only the findings obtained with ZeyEX treatment that were statistically significant (*p* < 0.01). In comparison with the sham group, serum levels of matrix metalloproteinases (MMP-3, MMP-9, and MMP-13) were found to be significantly elevated in the OA group (*p* < 0.001, *p* < 0.05, *p* < 0.001, respectively) (Fig. 4E–G). All four treatments demonstrated a significant suppression of MMP-3 levels (*p* < 0.05). MMP-9 was suppressed by ZeyEX, NPROC, and REJENERA at a significance level of *p*< 0.001, while it was suppressed by IBUPROFEN at a significance level of *p* < 0.05. The increase in MMP-13 levels induced by OA was significantly inhibited only by REJENERA treatment (*p* < 0.001). Serum levels of the enzyme tissue inhibitor of metalloproteinases-1 (TIMP-1) were found to be significantly elevated in rats suffering from OA in comparison with the sham group (*p* < 0.05) (see Fig. 4H). Treatment with ZeyEX (*p* < 0.01), NPROC (*p* < 0.05), and IBUPROFEN (*p* < 0.05) resulted in a significant reduction in TIMP-1 levels; however, the reduction in TIMP-1 levels after REJENERA treatment was not significant (*p* > 0.05) .

### The concentration of cytokines, IL-1β, IL-6, TNF-α, IFN-γ, IL-10 and IL-2, in synovial fluid

**Fig. 5:**
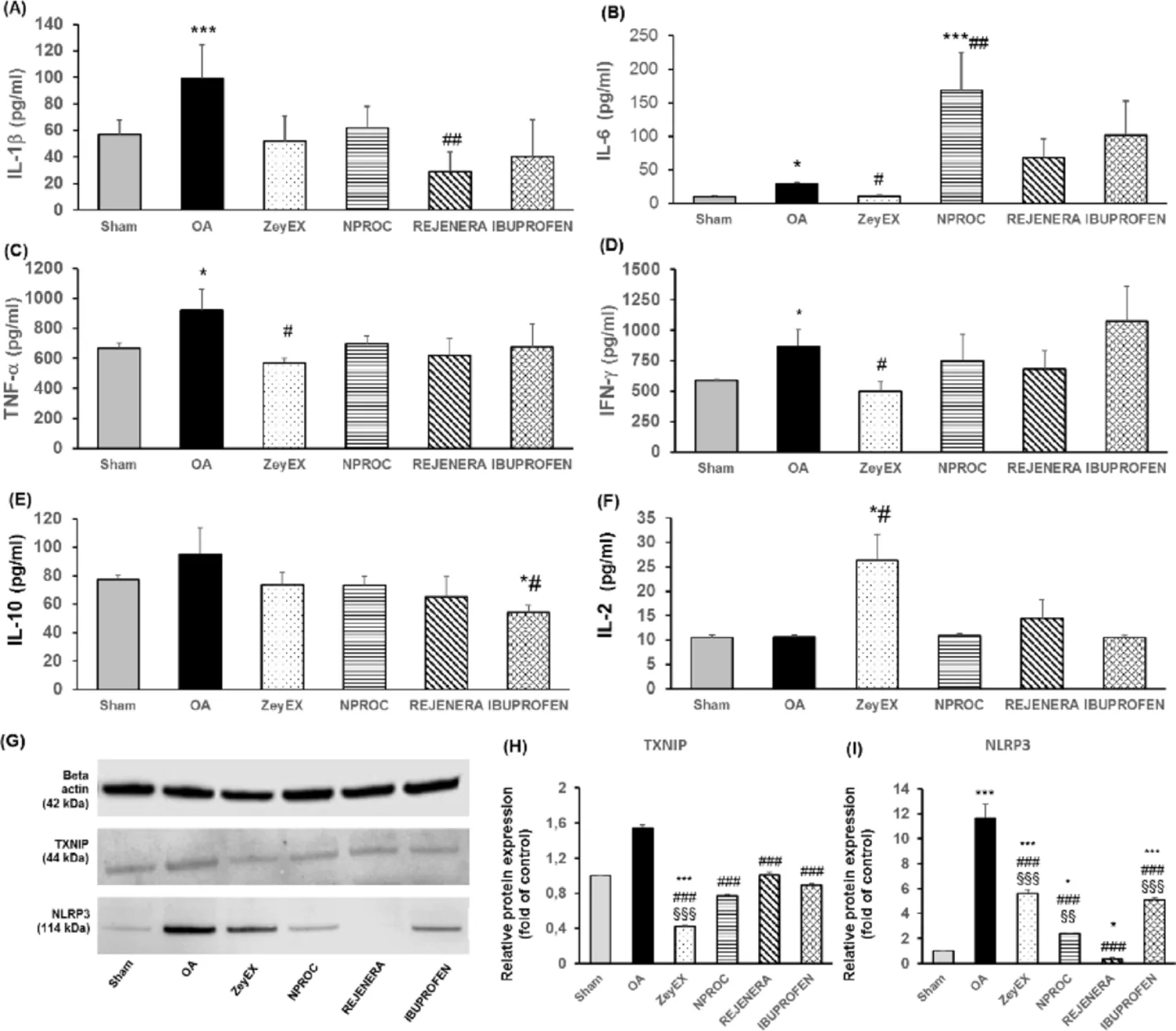
Changes in synovial fluid levels of (**A**) IL-1β, (**B**) IL-6, (**C**) TNF-α, (**D**) IFN-γ, (**E**) IL-10) and (**F**) IL-2 in Sham, osteoarthritis (OA),ZeyEX, NPROC, REJENERA and IBUPROFEN treatment groups. n = 6 for each experimental group. Cytokine concentrations were analyzed using the Kruskal–Wallis test followed by pairwise comparisons with Bonferroni correction. Data are expressed as mean ± SD. *p < 0.05, **p < 0.01, ***p < 0.001 *vs*. Sham; ^#^*p* < 0.05, ^##^*p* < 0.01, ^###^*p* < 0.001 *vs*. OA. TXNIP and NLRP3 inflammasome protein expressions in cartilage tissue were determined by Western blot analysis, and relative expression levels are shown in panel (**G**). Blots shown are representetive images from independent experiments. Quantitative values obtained by quantifying Western blot bands by densitometry are graphed for TXNIP (**H**) and NLRP3 (**I**), and were anayzed using one-way ANOVA followed by Tukey’s post hoc test. Actin levels were measured for the confirmation of equal protein loading. The sample size was n = 4 for each group. All results were represented as mean ± SEM from three independent experiments. *p < 0.05, **p < 0.01, ***p < 0.001 *vs*. Sham;  ^⋕^*p* < 0.05, ^⋕^^⋕^*p* < 0.01,  ^⋕^^⋕^^⋕^*p* < 0.001 *vs*. OA; ^§^*p* < 0.05, ^§^^§^*p* < 0.01, ^§^^§^^§^*p* < 0.001 *vs*. REJENERA

Multiplex analysis revealed significant differences in the concentrations of various inflammatory markers in synovial fluid samples from animals with knee OA. Compared with sham controls, untreated OA rats exhibited an approximately 1.5-fold increase in IL-1β levels (*p* < 0.05), together with elevated IL-6, TNF-α, and IFN-γ concentrations (*p* < 0.05), whereas no significant changes were observed in the anti-inflammatory cytokines IL-10 and IL-2 (Fig. 5A–F). ZeyEX treatment significantly reduced IL-6, TNF-α, and IFN-γ levels (*p* < 0.05); notably, TNF-α and IFN-γ concentrations decreased by approximately 35–40%, approaching the levels observed in sham animals, while IL-2 concentrations increased nearly 2.5-fold relative to untreated OA rats (*p* < 0.05). In contrast, NPROC treatment resulted in the highest IL-6 concentrations among all experimental groups (*p* < 0.01 *vs*. OA). REJENERA treatment markedly reduced IL-1β levels (*p* < 0.01), whereas IBUPROFEN treatment lowered IL-10 concentrations below those observed in sham animals (*p* < 0.05) and showed a tendency toward increased IFN-γ levels (Fig. 5A–F).

### TXNIP and NLRP3 protein expressions in joint cartilage

To further investigate the protective mechanism of our potential therapeutic products on OA-induced oxidative stress and inflammatory response, thioredoxin-interacting protein (TXNIP) and nucleotide-binding domain-like receptor protein 3 (NLRP3) were analyzed using Western blot. Quantification of TXNIP and NLRP3 protein expression levels in cartilage revealed an increase in OA (Fig. 5G). TXNIP upregulation was associated with increased protein expression of NLRP3. High ROS levels can dissociate TXNIP from thioredoxin and bind to activate NLRP3, which further leads to an inflammatory response (Choi and Park 2023). All treatments significantly reduced the expression of both TXNIP and NLRP3 in OA cartilage (*p* < 0.001 *vs*. OA)(Fig. 5G–I). Interestingly, ZeyEX reduced TXNIP expressions below the values detected in the Sham group (*p* < 0.001). Compared to other treatments, REJENERA demonstrated the most significant inhibition of the OA-induced increase in NLRP3 expression levels (*p* < 0.001 *vs*. OA; *p* < 0.05 *vs*. Sham) (Fig. 5G–I).

### Effects of ZeyEX, NPROC, REJENERA and IBUPROFEN on histomorphological changes: hematoxylin–eosin staining results

#### Knee joint structure and histology

**Fig. 6:**
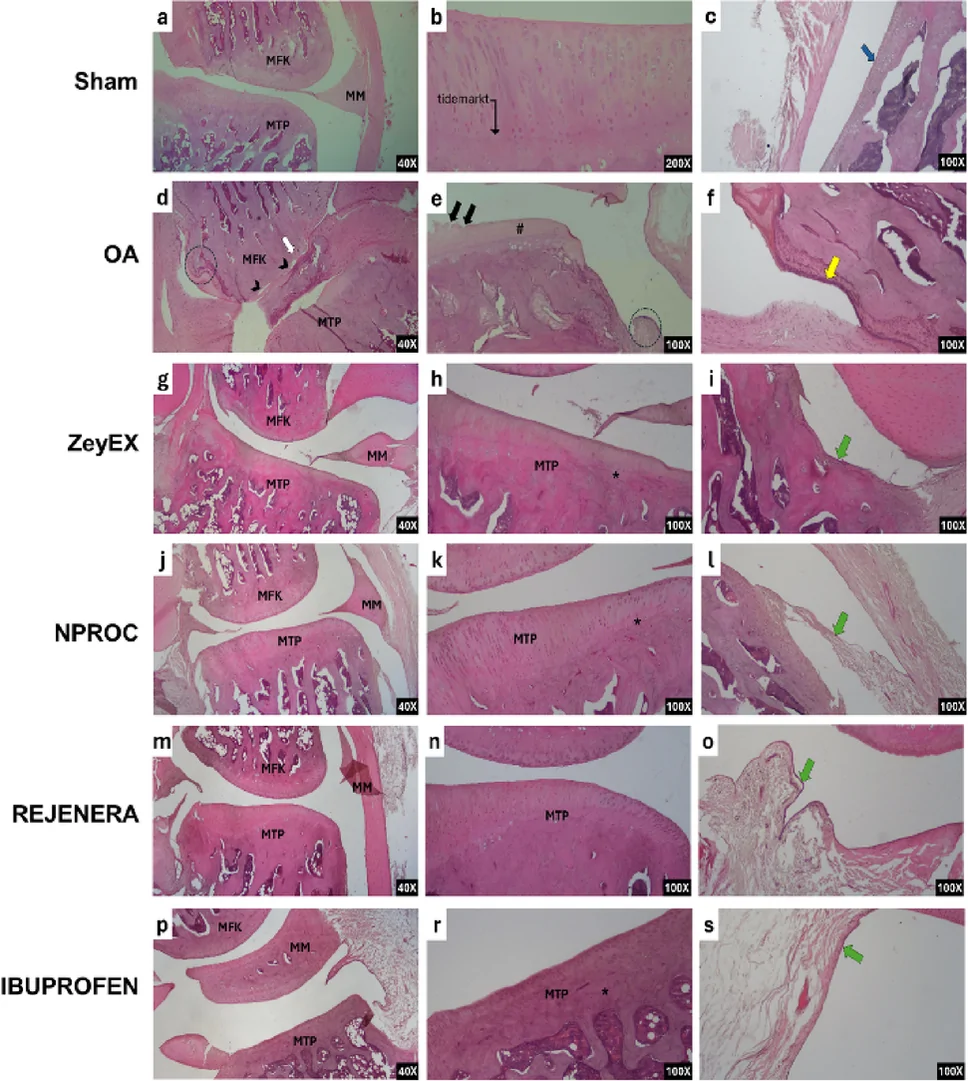
Representative histomorphological images of the articular cartilage of Sham (**a**–**c**), OA (**d**–**f**), ZeyEX (**g**–**i**), NPROC (**j**–**l**), REJENERA (**m**–**o**) and IBUPROFEN (*p*-s) groups (MFK: medial femoral condyle, MTP: medial tibial plateau, MM: medial meniscus, blue arrow: normal (1–2 cell thickness) synovium, white arrow: chondrocyte clustering, arrowhead: cartilage loss, circle: osteophyte formations, black arrow: fissure formations, #: large areas of degenerated chondrocytes in calcified cartilage, yellow arrow: inflammatory synovium, *: destructive tidemark, green arrow: near-normal (2–5 cell thickness) synovium) (H&E)

The femoral condyles and tibial plateaus of the knee joint were analysed using in vivo histopathological data. When the groups were evaluated using haematoxylin and eosin (H&E) staining, the following were observed in the sham group: an intact tidemark; intact cartilage structure; intact synovial structure; and intact matrix staining (Fig. 6a–c, blue arrow). In contrast, extensive cartilage degradation and destruction of the tidemark structure were observed in the OA group (Fig. 6d–f). Fissure formations were observed at varying levels on the articular cartilage surface (black arrow). Articular cartilage loss down to the subchondral bone was demonstrated in the OA group (arrowhead). Chondrocytes that did not produce matrix (*), clusters of chondrocytes (white arrow), and large areas of degenerated chondrocytes in the calcified cartilage (**#**) were present in numerous areas of the OA group. Osteophyte formation (circles) was also noted in the OA group. When the synovium was evaluated, areas of inflammation (yellow arrow) reaching 8–10 cell layers in size were observed in the OA groups (Fig. 6d–f). In the ZeyEX group, while fibrillation and fissures were evident in the condylar and plateau cartilage, the overall cartilage structure was found to be partially intact. Furthermore, the synovium exhibited the presence of inflammatory cells, measuring 3–5 cells in thickness (Fig. 6g–i). In the NPROC group, there was evidence of localized cartilage degeneration; however, the overall integrity of the joint structure and staining intensity were significantly superior. In certain regions, the degenerative changes to the cartilage appeared to be destructive, while the synovial inflammation was minimal (Fig. 6j–l). In the REJENERA group, degenerative changes in cartilage and fissure areas were identified in the condylar and plateau cartilages. However, the overall structure of the joints, bones, cartilage, and surrounding tissues remained largely intact (Fig. 6m–o). In the IBUPROFEN group, degenerative cartilage areas and a small number of fissures were observed, but the cartilage matrix was well stained. However, the presence of impaired border areas was a notable observation. The presence of synovial inflammation was found to be minimal (Fig. 6p–s).

#### Fissure index findings

The OA group demonstrated the highest FI values (median: 1.22), indicating pronounced surface fibrillation and cartilage degeneration. In comparison, all treatment groups demonstrated lower FI values, although the magnitude of improvement varied. In comparison to the OA group, median FI values exhibited a decrease of approximately 5.7% in the ZeyEX group (1.15), 10.7% in the NPROC group (1.09), 14.8% in the REJENERA group (1.04), and 13.9% in the IBUPROFEN group (1.05) (Table 2). Among the treatment groups, REJENERA and IBUPROFEN demonstrated the greatest reduction in FI, with values approaching those observed in the sham group (1.01), suggesting a stronger protective effect against cartilage surface deterioration. Statistical analysis demonstrated that the FI value of the sham group was significantly lower than those of the OA (*p* < 0.001), ZeyEX (*p* < 0.001), NPROC (*p* < 0.001), and IBUPROFEN (*p* = 0.004) groups, whereas no significant difference was detected between the sham and REJENERA groups (*p* = 0.352). In comparison with the OA group, the reductions observed in the ZeyEX and NPROC groups were not statistically significant (*p* = 1.000 and *p* = 0.080, respectively). In contrast, the REJENERA and IBUPROFEN groups demonstrated significantly lower FI values in comparison to the OA group (both *p* < 0.001), indicating a marked attenuation of cartilage surface damage (Table 2).

**Table 2:** Descriptive data of articular cartilage fissure index (FI) for all experimental groups.

	FI (L/W)	
	Mean ± SD	Median (Min–Max)
Sham	1.01 ± 0.01	1.01 (1.00–1.04)^#^
OA	1.21 ± 0.09	1.22 (1.05–1.38)*
ZeyEX	1.16 ± 0.06	1.15 (1.08–1.25)*
NPROC	1.09 ± 0.04	1.09 (1.04–1.23)*
REJENERA	1.05 ± 0.02	1.04 (1.00–1.10)^#^
IBUPROFEN	1.06 ± 0.02	1.05 (1.04–1.10)*^#^

Using H&E staining, fractures were evaluated in terms of depth, branching, and vertical extension. To quantitatively describe the degree of superficial cracking, the fissure index (FI) was calculated as follows: FI = length of cartilage surface (L)/width of the measured area (W) (Pastoureau et al. 2010). *p < 0.05 *vs*. Sham; ^#^*p* < 0.05 *vs*. OA. Multi-group variations were analyzed via Kruskal–Wallis, and pairwise comparisons via Bonferroni-corrected Mann–Whitney U test

#### Osteophyte score findings

Osteophytes of various size were observed in the OA group. The osteophyte score exhibited a statistically significant increase in the OA group in comparison to the sham group (*p* = 0.009) (Table 3). There was no statistically significant difference (*p* > 0.05) between the joints in the sham group and the active treatment groups, indicating that the treatments were successful in preventing the progression of bone spurs. These findings indicate a successful induction of OA, and reveal that the treatments reduce or prevent the growth of bone spurs (osteophytes) compared to the untreated condition.

**Table 3:** Osteophyte scores were assessed during histological evaluation to indicate the formation of bone spurs (osteophytes), a hallmark of OA

Grade	Osteophyte formation n (%)					
	Sham	OA	ZeyEX	NPROC	REJENERA	IBUPROFEN
0	19 (95)	11 (55)	17 (85)	17 (85)	15 (75)	16 (80)
1	1 (5)	1 (5)	2 (10)	2 (10)	5 (25)	3 (15)
2	–	8 (40)	1 (5)	1 (5)	–	1 (5)
Median (Min–Max)	0 (0–1)^#^	0 (0–2)*	0 (0–2)^#^	0 (0–2)#	0 (0–1)^#^	0 (0–2)^#^

In H&E (Hematoxylin and Eosin) stained sections, osteophytes were graded on a semi-quantitative scale ranging from 0 to 2, according to size, tissue maturity, and joint margin involvement. Frequency (n), percentage (%), and median (minimum–maximum) values of osteophyte scores in all experimental groups. 0: none, 1: small, 2: large. *p < 0.05 vs. Sham; ^#^*p* < 0.05 vs. OA. Multi-group variations were analyzed via Kruskal–Wallis, and pairwise comparisons via Bonferroni-corrected Mann–Whitney U test

#### Synovial inflammation score findings

The severity of synovial inflammation, which is indicative of synovial lining thickness and inflammatory cell infiltration, was scored. The mean values were found to be significantly higher in the OA group compared to the sham group (*p* < 0.001). The mean score in the IBUPROFEN group was significantly lower than in the OA group (*p* = 0.015). The inflammation scores demonstrated a downward trend in the REJENERA, ZeyEX, and NPROC groups. While this reduction was statistically significant when compared to the sham group (*p* = 0.001, 0.013, and 0.047, respectively), it was only statistically significant in the IBUPROFEN group when compared to the OA group (*p* = 0.015) (Table 4).

**Table 4:** Synovitis scores were calculated using a semi-quantitative scoring system that assessed synovial lining hyperplasia, inflammatory infiltration, and stromal fibrosis on H&E-stained sections (0: 1–2 cell thickness, 1: 3–5 cell thickness, 2: 5–8 cell thickness, 3: > 9 cell thickness)

Grade	Synovial inflammation n (%)					
	Sham	OA	ZeyEX	NPROC	REJENERA	IBUPROFEN
0	15 (75)	–	5 (25)	6 (30)	3 (15)	8 (40)
1	5 (25)	9 (45)	8 (40)	8 (40)	8 (40)	8 (40)
2	–	6 (30)	6 (30)	5 (25)	8 (40)	4 (20)
3	–	5 (25)	1 (5)	1 (5)	1 (5)	–
Median (Min–Max)	0 (0–1)^#^	2 (1–3)*	1 (0–3)*	1 (0–3)*^#^	1 (0–3)*	1 (0–2)*^#^

Table shows frequency (n), percentage (%), and median (minimum–maximum) values of synovial inflammation degrees in all groups. *p < 0.05 vs. Sham; ^#^*p* < 0.05 vs. OA. Multi-group variations were analyzed via Kruskal–Wallis, and pairwise comparisons via Bonferroni-corrected Mann–Whitney U test

### Effects of ZeyEX, NPROC, REJENERA and IBUPROFEN on bone and cartilage histology and destruction: safranin O-fast green staining results

#### General histopathological assessments and OARSI findings

**Fig. 7:**
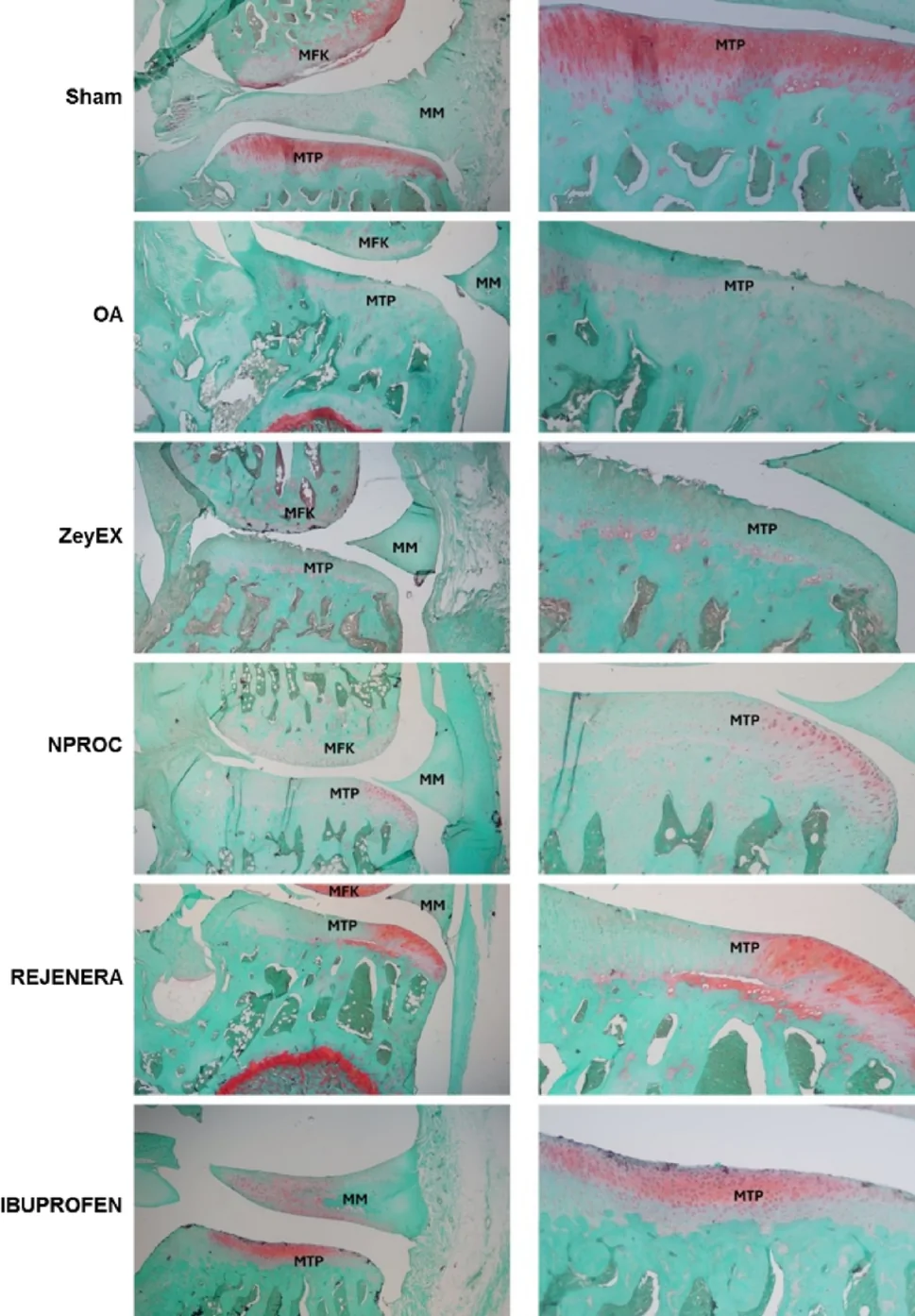
Representative images of the articular cartilage of all groups in the sagittal plane section with safranin-O fast green staining. MFK: Medial femoral condyle, MTP: Medial tibial plateau, MM: Medial meniscus. Magnification, left column: x40; right column: x100

The cartilage structure and matrix staining were observed to be normal in the sham group. The articular cartilage of the tibial plateau and femoral condyle exhibited a smooth surface in this group. Conversely, degeneration including fibrillation and cartilage loss was evident in the condyles and plateaus of the OA group. Increased tibial subchondral bone areas were clearly evident in the OA group. In comparison with the OA group, there was significantly less fibrillation, cartilage loss with better matrix staining in the condyles and plateaus of the treatment groups, particularly in the REJENERA and IBUPROFEN groups (Fig. 7). A subsequent evaluation of the OARSI scores of the knee joints revealed that the scores in the OA and treatment groups were significantly higher than in the sham group (*p* < 0.05). In the sham group, 81.3% (n = 13) of the sham samples scored 0 and 18.8% (n = 3) scored 1. In contrast, OA induction caused a severe pathological shift, with 68.8% (n = 11) of the untreated OA cohort progressing to advanced degenerative stages with scores between 4 and 6. However, it was observed that all treatment modalities resulted in a decrease in OARSI scores when compared to the OA group, thereby effectively preventing progression into the terminal stage (Score 6). This decrease was statistically significant in the REJENERA and IBUPROFEN groups (*p* = 0.009 and 0.006, respectively). Specifically, both the REJENERA and IBUPROFEN groups exhibited a restriction of advanced cartilage destruction (scores ≥ 4) to a mere 6.3% (n = 1), signifying a 90.8% relative reduction in severe lesion frequency in comparison to the OA group. Concurrently, the NPROC and ZeyEX groups exhibited moderate mitigation, reducing severe degeneration (scores 4–5) to 25.0% (n = 4) and 37.6% (n = 6), respectively (Table 5).

**Table 5:** Frequency (n), percentage (%), and median (minimum–maximum) values of OARSI scores in all groups.

Score	OARSI n (%)					
	Sham	OA	ZeyEX	NPROC	REJENERA	IBUPROFEN
0	13 (81.25)	–	–	–	–	–
1	3 (18.75)	–	2 (12.5)	2 (12.5)	4 (25.0)	5 (31.25)
2	–	2 (12.5)	2 (12.5)	6 (37.5)	4 (25.0)	6 (37.5)
3	–	3 (18.75)	6 (37.5)	4 (25.0)	7 (43.75)	4 (25.0)
4	–	3 (18.75)	3 (18.75)	2 (12.5)	1 (6.25)	1 (6.25)
5	–	6 (37.5)	3 (18.75)	2 (12.5)	–	–
6	–	2 (12.5)	–	–	–	–
Median (Min–Max)	0 (0–1)^#^	4.5 (2–6)*	3 (1–5)*	2.5 (1–5)*	2.5 (1–4)*^#^	2 (1–4)*^#^

*p < 0.05 *vs*. Sham; ^#^*p* < 0.05 *vs*. OA. Multi-group variations were analyzed via Kruskal–Wallis, and pairwise comparisons via Bonferroni-corrected Mann–Whitney U test

#### Cartilage thickness findings

When the total thickness of hyaline and calcified cartilage in the knee joint was evaluated, it was found that the cartilage thickness in the OA group was statistically significantly decreased compared to the sham group (*p* < 0.001). Both the ZeyEX and NPROC groups showed no significant difference when compared directly to the OA group (*p* = 1.000 and 1.000, respectively) (Table 6). However, both REJENERA and IBUPROFEN displayed a statistically significant increase in cartilage thickness compared to the OA group (*p* = 0.002 and 0.029, respectively) (Table 6). The results indicate that while the ZeyEX and NPROC interventions failed to restore or protect cartilage thickness from OA-related degeneration, both the REJENERA treatment and the IBUPROFEN regimen demonstrated promising cartilage-protective or regenerative properties in this model.

**Table 6:** Articular cartilage thickness alterations for all experimental groups

	Cartilage thickness (µm)	
	Mean ± SD	Median (Min–Max)
Sham	401.21 ± 38.17	406.00 (298.00–547.00)^#^
OA	205.33 ± 93.93	203.50 (37.00–411.00)*
ZeyEX	221.49 ± 86.20	226.50 (57.00–436.00)*
NPROC	235.86 ± 95.51	239.50 (38.00–425.00)*
REJENERA	270.43 ± 107.20	288.00 (53.00–522.00)*^#^
IBUPROFEN	260.36 ± 113.31	266.50 (36.00–561.00)*^#^

Thickness of articular cartilage were measured by histology of knee joints. *p < 0.05 *vs*. Sham; #*p* < 0.05 *vs*. OA. Multi-group variations were analyzed via Kruskal–Wallis, and pairwise comparisons via Bonferroni-corrected Mann–Whitney U test

#### Proteoglycan loss findings

**Fig. 8:**
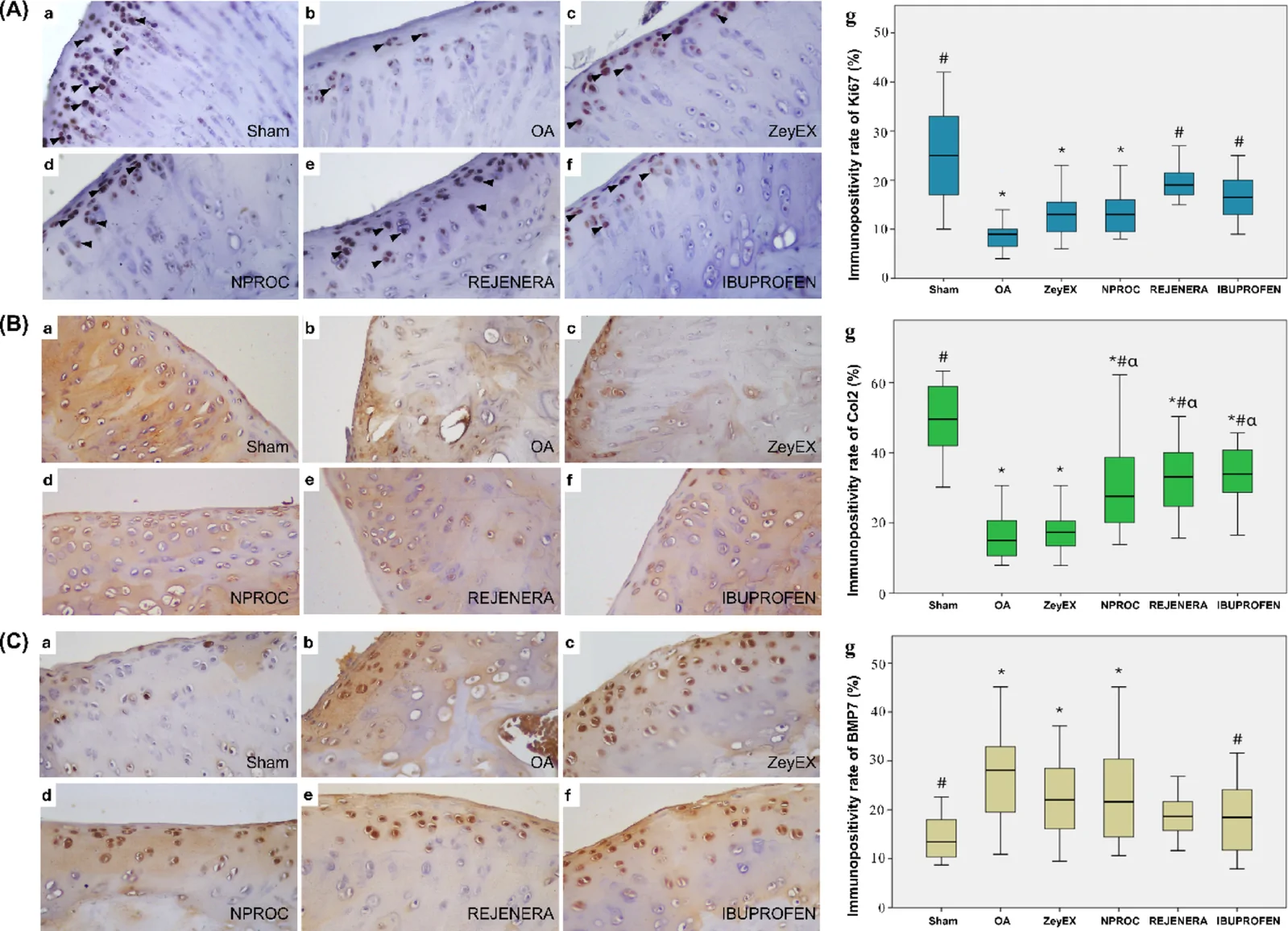
Representative micrographs of (**A**) Ki67, ** (B) **collagen-II (Col2) and (**C**) BMP7 (Bone Morphogenetic Protein 7) immunopositivity densities in the articular cartilage of Sham (**a**), OA (**b**), ZeyEX (**c**), NPROC (**d**), REJENERA (**e**) and IBUPROFEN (**f**) groups.  The findings of quantitative anaysis are shown in the (**g**) sub-panels of panels (A), (B) and (C) for each antibody.  Arrows indicate positively stained cells (DAB-hematoxylin, x400). Data were analyzed using the Bonferroni-corrected Mann-Whitney test. *p < 0.05, **p < 0.01, ***p < 0.001 *vs*. Sham; ^#^*p* < 0.05, ^##^*p* < 0.01, ^###^*p* < 0.001 *vs*. OA; ^αα^p < 0.01, ^ααα^p < 0.01 *vs*. ZeyEX

Proteoglycan loss was significantly higher in the OA and treatment groups than in the sham group (*p* < 0.001) (Fig. 8C). All treatment groups (ZeyEX, NPROC, REJENERA and IBUPROFEN) showed a statistically significant reduction in proteoglycan loss compared to the OA group (*p* = 0.004, 0.002, < 0.001 and < 0.001 respectively). Statistical data showed that proteoglycan loss was reduced in all treatment groups, but the significance was higher in the REJENERA and IBUPROFEN groups (Table 7).

**Table 7:** Frequency (n), percentage (%), and median (minimum–maximum) values of proteoglycan loss degrees in all experimental groups according to the Safranin O-Staining method of cartilage

	Proteoglycan loss n (%)					
Grade	Sham	OA	ZeyEX	NPROC	REJENERA	IBUPROFEN
0	18 (90)	–	–	–	–	–
1	2 (10)	–	3 (15)	4 (20)	8 (40)	4 (20)
2	–	1 (5)	8 (40)	7 (35)	6 (30)	8 (40)
3	–	8 (40)	6 (30)	7 (35)	6 (30)	7 (35)
4	–	11 (55)	3 (15)	2 (10)	–	1 (5)
Median (Min–Max)	0 (0–1)#	4 (2–4)*	2 (1–4)*^#^	2 (1–4)*^#^	2 (1–3)*^#^	2 (1–4)*^#^

0: normal, 1:minimum, 2:mild, 3:moderate; 4:severe. *p < 0.05 vs. Sham; ^#^*p* < 0.05 *vs*. OA. Multi-group variations were analyzed via Kruskal–Wallis, and pairwise comparisons via Bonferroni-corrected Mann–Whitney U test

### Immunohistochemical staining results

#### Ki-67 findings

The percentages of Ki67 immunostaining in the articular cartilage were determined as 25.13 ± 9.39 in the sham group; 9.04 ± 3.74 in the OA group; 12.88 ± 5.06 in the ZeyEX group; 13.17 ± 4.18 in the NPROC group; 18.38 ± 5.64 in the REJENERA group; and 16.58 ± 5.21 in the IBUPROFEN group. Compared to the sham group, a decrease in the number of positive cells in the medial tibial plateau regions was observed in the other groups (Fig. 8A). Ki67 immunopositivity decreased in all experimental groups compared to the sham group; the lowest value was observed in the OA group (*p* < 0.001) (Fig. 8A). Treatment with ZeyEX, and NPROC induced partly amelioration in the number of Ki67 positive cells, but the mean values obtained were sitill significantly lower compared to sham group (*p* < 0.05). Treatment with REJENERA and IBUPROFEN significantly improved the number of Ki67 positive cells; the mean immunopositivity rates (%) obtained from these groups were not statistically significant compared to the control group (*p* = 1.000 and 0.187, respectively) (Fig. 8A).

#### Collagen-II (Col-2) findings

The percentages of Col-2 immunopositivity detected in the medial tibial plateau regions of the articular cartilage were 48.94 ± 10.98 in the sham group, 15.90 ± 6.21 in the OA group, 17.69 ± 6.45 in the ZeyEX group, 30.50 ± 12.56 in the NPROC group, 32.59 ± 9.05 in the REJENERA group, and 33.02 ± 8.22 in the IBUPROFEN group. Relative to the sham group, Col-2 immunopositivity was reduced in the OA, ZeyEX, NPROC, REJENERA, and IBUPROFEN groups, with all comparisons reaching statistical significance (*p* < 0.001, *p* < 0.001, *p* = 0.001, *p* = 0.021, and *p* = 0.029, respectively). Col-2 uptake was lowest in the OA group. When the OA group was compared with the other groups, the difference between the ZeyEX group was not statistically significant (*p* = 1.000). While the mean value obtained from ZeyEX group did not differ from that in the OA group (*p* = 1.000), the treatments with NPROC, REJENERA, and IBUPROFEN exhibited significantly higher Col-2 positivity compared to OA and ZeyEX (*p* = 0.001, *p* < 0.001, and *p* < 0.001, respectively) (Fig. 8B).

#### BMP7 findings

The percentages of Bone morphogenetic proteins-7 (BMP7) immunopositive stained cells in the medial tibial plateau regions of the articular cartilage were 14.54 ± 5.51 in the sham group, 27.10 ± 8.39 in the OA group, 21.97 ± 8.24 in the ZeyEX group, 23.01 ± 8.93 in the NPROC group, 18.98 ± 4.11 in the REJENERA group, and 18.51 ± 7.02 in the IBUPROFEN group. BMP7 positivity rates in articular cartilage were found to be significantly higherin the untreated- and treated-OA groups compared to the sham group. These increases in BMP7 immunopositivity in the OA, ZeyEX, and NPROC groups were statistically significant (*p* < 0.001, *p* = 0.009, and *p* = 0.003, respectively). No statistically significant difference was found in the positivity rates between the sham group and the REJENERA and IBUPROFEN groups (*p* = 0.222 and *p *= 0.702, respectively). Relative to the OA group, BMP7 positivity was significantly lower in the sham and IBUPROFEN groups (*p* < 0.001 and *p* = 0.014, respectively) (Fig. 8C).

#### iNOS/NOS2 findings

**Fig. 9:**
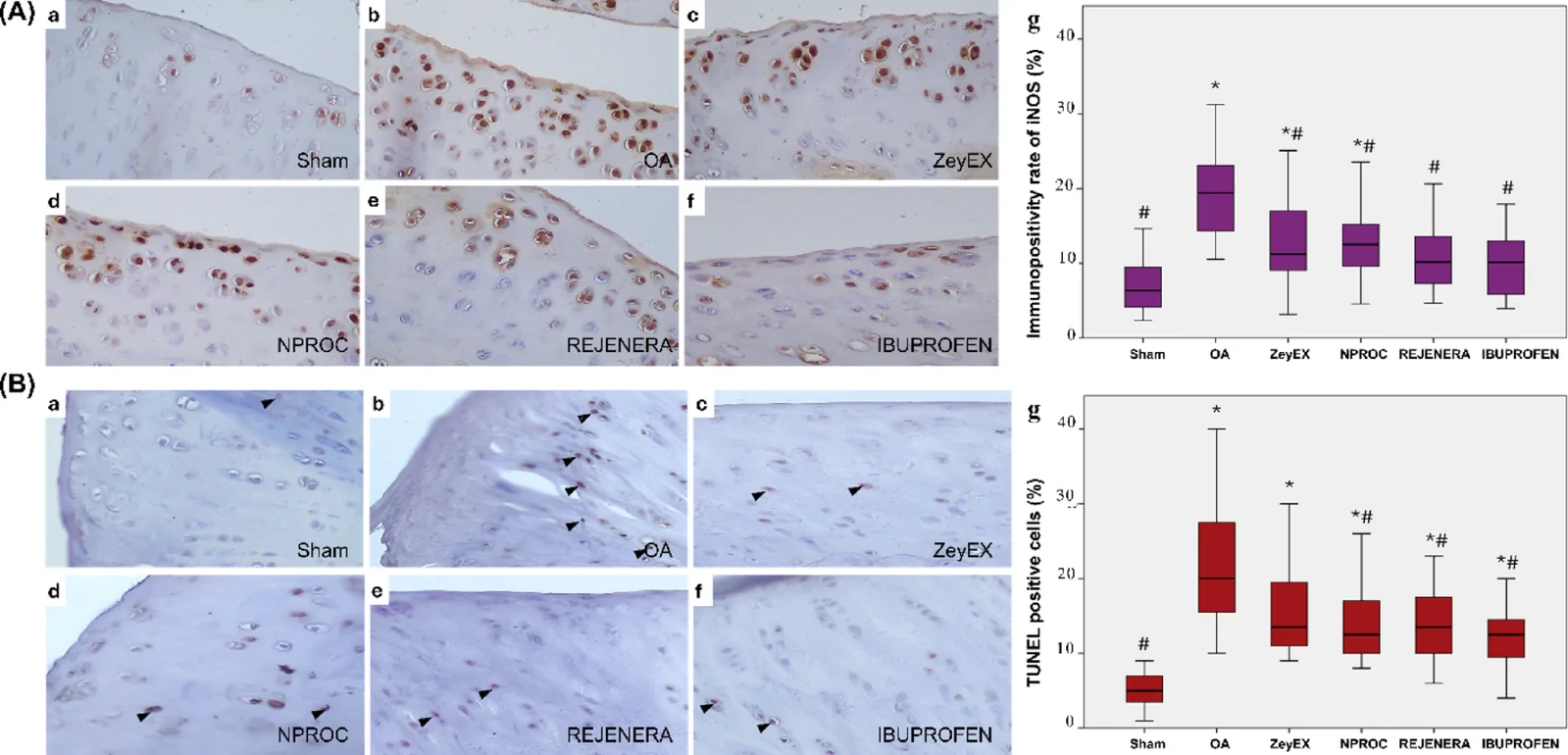
Representative micrographs of (**A**) iNOS (inducible nitric oxide synthase) immunostaining, and (**B)** TUNEL positive cell densities in the articular cartilage of Sham (**a**), OA (b), ZeyEX (**c**), NPROC (**d**), REJENERA (**e**) and IBUPROFEN (**f**) groups. Arrows indicate positively stained cells (DAB-hematoxylin, x400). Quantitative analyses of iNOS immunopositivity and TUNEL-positive cell rates are shown in (**g**) sub-panels. Arrows indicate positevely stained cells  (DAB-hematoxylin, x400). Data were analyzed using the Bonferroni-corrected Mann-Whitney test. *p < 0.05, **p < 0.01, ***p < 0.001 *vs*. Sham; ^#^*p* < 0.05, ^##^*p* < 0.01, ^###^*p* < 0.001 *vs*. OA

The percentages of iNOS immunopositive stained cells in the medial tibial plateau regions of the articular cartilage were 6.89 ± 3.33 in the Sham group; 19.40 ± 6.12 in the OA group; 13.03 ± 6.23 in the ZeyEX group; 12.77 ± 5.07 in the NPROC group; 11.08 ± 5.05 in the REJENERA group; and 9.59 ± 3.94 in the IBUPROFEN group. iNOS immunopositivity was increased in all experimental groups relative to the sham group, with the highest positivity rate observed in the OA group (Fig. 9A). Significant increases were detected in the OA, ZeyEX, and NPROC groups compared with sham (*p* < 0.001, *p* = 0.009, and *p* = 0.002, respectively), whereas the REJENERA and IBUPROFEN groups did not differ significantly from sham (*p* = 0.082 and *p* = 0.743, respectively). Compared with the OA group, all remaining groups showed significantly lower iNOS immunopositivity (*p* < 0.05) (*p* < 0.05) (Fig. 9A).

#### TUNEL findings

The percentages of TUNEL-positive cells in articular cartilage were 5.04 ± 2.37 in the sham group; 21.21 ± 7.59 in the OA group; 15.83 ± 6.60 in the ZeyEX group; 13.92 ± 5.35 in the NPROC group; 13.46 ± 4.91 in the REJENERA group; and 11.88 ± 4.08 in the IBUPROFEN group.

TUNEL positivity was increased in all experimental groups compared with the sham group, with the highest level observed in the OA group. The increases detected in the ZeyEX, NPROC, REJENERA, and IBUPROFEN groups were statistically significant relative to the sham group (*p* < 0.001). Compared with the OA group, TUNEL positivity was reduced in all treatment groups; however, this reduction did not reach statistical significance in the ZeyEX group (*p* = 0.467). In contrast, the NPROC, REJENERA, and IBUPROFEN groups showed significantly lower numbers of TUNEL-positive cells than the OA group (*p* = 0.038, *p* = 0.027, and *p* = 0.002, respectively) (Fig. 9B).

## Discussion

The present study evaluated the therapeutic potential of REJENERA, a novel multi-component bioflavonoid-based formula, consisting primarily olive leaf product, ZeyEX together with additional cartilage-acting compounds, by comparing it with ZeyEX alone, its nanoencapsulated form NPROC, and the reference drug IBUPROFEN in a monoiodoacetate (MIA)-induced rat model of osteoarthritis (OA).

MIA injection produced a consistent OA-like phenotype characterized by joint swelling, macroscopic joint damage, cartilage surface disruption, proteoglycan loss, increased OARSI score, synovial inflammation, increased catabolic mediators, altered cartilage anabolic factors, increased TXNIP/NLRP3 expression, increased iNOS immunopositivity, and enhanced chondrocyte apoptosis. In this experimental model of OA, treatment interventions have demonstrated efficacy in arresting disease progression. These experimental treatments successfully mitigate cartilage degradation by mainly inhibiting catabolic mediators and inflammation. In this context, a comparison of the treatments revealed that ZeyEX exhibited a more comprehensive response in OA rats in terms of inhibition of oxidative parameters and inflammatory cytokines, while REJENERA specifically demonstrated effective regulation of NLRP3 expression and histomorphology. The findings indicate that the combined components of REJENERA interact with multiple pathological pathways in OA, which may provide a rationale for the broader effect profile observed across the parameters examined.

The anti-OA efficacy of REJENERA can be attributed to the multi-target pharmacological effects of ZeyEX, an olive leaf extract that is rich in oleuropein phenolic content, and its phenolic components, which are present in the formula. The anti-OA effects and mechanisms of action of these components have been demonstrated in cell culture studies (Elmazoglu et al. 2020a, b). The REJENERA formulation also contains other olive leaf phenolics such as luteolin and quercetin, as well as other components that have been reported to have some anti-OA properties. Indeed, the anti-OA effects of olive leaf extracts have been documented in a limmited numberof articles (Szychlinska et al. 2019; Horcajada et al. 2022), however no studies have been reported in the MIA model of OA. In view of the findings of the present study,  it can be concluded that the primary mechanism of the anti-OA effect of REJENERA and ZeyEX components is most likel to be the attenuation of inflammatory, oxidative/nitrosative, and catabolic signaling, particularly through modulation of the TXNIP/NLRP3 inflammasome axis. The process of MIA-induced OA and its progression are the results of a complex interply between various factors, including the actions of the chondrocytes, the synovium, subchondral bone, inflammatory cytokines, lipid peroxidation, oxidative stress, nitric oxide signaling, and enzymes that facilitate the degradation of the extracellular. These mediators are closely related to chondrocyte dysfunction, extracellular matrix degradation, synovial inflammation, and apoptotic cell death in experimental OA (Lakshmanan et al. 2020; Lu et al. 2024). Treatment-associated reductions in inflammatory mediators, MMPs expression, and iNOS immunopositivity suggest that the tested agents may act mainly by reducing the inflammatory-catabolic environment within the joint. Consequently, the observed improvements in cartilage matrix markers, cartilage thickness, proteoglycan retention, and apoptosis should be interpreted as predominantly downstream consequences of diminished inflammatory, oxidative, and catabolic stress, as opposed to being interpreted as direct evidence of cartilage regeneration or disease modification.

ZeyEX, theprimary constituent of both NPROC and REJENERA, effectively counteracts the increases in inflammatory markers, such as IL-6, TNF-α, and IFN-γ levels, in joint fluid, and IL-6, MMP-3, and MMP-9 levels in serum, induced by OA. It is noteworthy that ZeyEX prevents the OA-mediated increase in IL-6 in both serum and synovial fluid.Conversely, the synovial fluid of animals with OA exhibited a statistically significant increase in IL-6 levels in response to NPROC. Recent studies suggest that neutralization of IL-6 signaling is a potential target for modification of OA diseases (Eitner et al. 2024). In addition, ZeyEX was the only product that had a statistically significant effect in reducing lipid peroxidation (LPO), which is another important sign that it reduces inflammatory cytokine release by preventing oxidative stress and regulates stress-related pathways in cells (Zubavlenko et al. 2021). In accordance with these satisfactory findings, IL-1β is significantly inhibited in synovial fluid exclusively by REJENERA. IL-1β has been identified as a central playerin the OA pathological process (Zhang et al. 2021), predominantly through its capacity to enhance the synthesis of proteolytic molecules (MMPs) and upregulate other inflammatory mediators (Wang and He 2018). Consistent with this, IL-1β treatment has been reported to significantly increase iNOS expression and inhibit Col-2 in OA chondrocytes (He et al. 2018). the present study demonstrated that, cartilage iNOS and serum MMPs were elevated in rats suffering from OA. As a free radical, NO has been shown to trigger the release of MMPs and to promote apoptosis in OA (Del Carlo and Loeser 2002). All treatment groups demonstrated a significant reductions in MMP-3 and MMP-9 levels when compared to the OA group. However, the OA-induced increase in MMP-13 was only reduced in REJENERA-treated animals, and this was achieved without significantly affecting TIMP-1 levels. MMP-13, the primary collagenase involved in OA pathology (Hu and Ecker 2021), cleaves Col-2 and is normally inhibited by TIMP-1 (Burrage and Brinckerhoff 2007). Under normal conditions, TIMPs bind to active MMPs, forming an inactive complex; in OA, an imbalance in the TIMP/MMP ratio leads to continuous matrix degradation (Cabral-Pacheco et al. 2020) and inhibition of Col-2, the predominant collagen in hyaline articular cartilage (Henrotin et al. 2014). The ability of REJENERA to reduce MMP-13 and iNOS without suppressing TIMP-1 is a noteworthy finding that distinguishes its profile from the other treatments examined. Overall,the biochemical evidence suggests that the modulation of inflammatory and matrix-degrading mediators is a contributing factor to the observed histological preservation.

Recent studies have demonstrated that the inhibition of TXNIP can serve as a therapeutic strategy to mitigate IL-1β-induced inflammation in articular cartilage (Xu et al. 2023; Cao et al. 2025). In the present study, ZeyEX was identified as the most efficacious product in reducing OA-induced TXNIP expression, lowering its expression level below that of the sham group. Other treatments also significantly inhibited TXNIP expression. A study by Altahla and Tao (2024) found that upregulation of TXNIP was associated with increased protein expression of NLRP3 in OA cartilage (Altahla and Tao 2024). When treatments were evaluated for their inhibitory effects on NLRP3 protein expression, REJENERA showed the most pronounced reduction in comparison with the OA group. TXNIP has been posited as a potential therapeutic target in OA given its role as a potential regulatory and functional factor in bone and cartilage metabolism (Jiang et al. 2022). These findings suggest that modulation of the TXNIP/NLRP3 axis represents a primary mechanism through which ZeyEX and REJENERA exert their anti-OA effects, with ZeyEX acting predominantly at the TXNIP level and REJENERA at the downstream NLRP3 level.

A further noteworty finding from our study is that synovial IL-2 levels in rats treated with ZeyEX were significantly higher compared to the sham group and the OA group. The afforementinoed effect was not observed in any of the other treatments. The regulatory effects of ZeyEX on IL-2 can be considered positive, given that previous studies have reported that low-dose IL-2 supplementation has broad therapeutic potential in the treatment of some inflammatory diseases, including OA (Graßhoff et al. 2021). IL-2 has been demonstrated to suppress increased osteoclastic activity and osteoclastogenesis in OA (Sun et al. 2020). Consequently, the augmented synovial IL-2 level subsequent to ZeyEX treatment may signify a regulatory anti-inflammatory immune response, thus necessitating further investigation of the molecular effects of olive leaf polyphenols in OA. On the other hand, NPROC is a nanoencapsulated form of olive leaf extract based on eggshell membrane, and is not a constituent of the REJENERA formulation. NPROC, whose oral bioavailability is potentially increased thanks to nanoencapsulation technology (Bayraktar et al. 2021), demonstrated ZeyEX-like effects in some parameters in this study. Nonetheless, the NPROC also resulted in a significant increase in IL-6 levels, a phenomenon that remained unexplained by an anti-OA effect. In fact, eggshell membrane,which is advocated as a functional component in the treatment of joint and connective tissue disorders due to its proven efficacy in relieving joint pain and stiffness, may affect the increase in IL-6 levels, as has been previously demonstrated (Yu et al. 2024). IBUPROFEN significantly reduced synovial IL-10 levels, even below those observed in the sham group. The cartilage-protective cytokine IL-10 (Guo et al. 2023) reduces TNF-α production and OA-associated TNF-α-mediated events. A body of research has identified that decreased localized IL-10 concentrations have a role in the exacerbation of a risk factors associated with osteophyte progression, joint space narrowing, and cartilage degeneration (Barker et al. 2021). This finding underscores a possible unfavorable outcome arising from IBUPROFEN’s mechanism of action on synovial immune equilibrium within the context of OA.

The immunohistochemical and TUNEL findings provide further evidence to support the hypothesis that reduced inflammatory-catabolic stress was accompanied by improved cartilage-cell homeostasis. MIA-induced OA reduced type II collagen immunopositivity and increased TUNEL-positive chondrocytes, indicating matrix disruption and apoptosis. Treatment-associated increases in type II collagen immunopositivity and reductions in TUNEL and iNOS immunopositivity in selected groups suggest preservation of cartilage matrix and chondrocyte viability due to reduced inflammatory and nitrosative stress. Bone morphogenetic proteins (BMPs), which are members of the transforming growth factor-beta (TGF-β) superfamily, have been identified as key regulators of cartilage homeostasis and repair (Daans et al. 2008). BMP-7 regulates chondrocyte metabolism by stimulating the synthesis, renewal, and retention of matrix molecules, and has demonstrated chondroprotective properties in animal models of OA (Whitty et al. 2022). In the present study, the expression of BMP-7 in OA cartilage was enhanced in animals treated with REJENERA in comparison to the OA group. Ki67, a nuclear protein expressed during cell proliferation, serves as an inflammatory arthropathy biomarker indicating the severity of osteoarthritis (OA), with lower Ki67 scores reflecting decreased mitotic activity (Pessler et al. 2008). Ki67 findings revealed a significantly reduced proliferative response in OA cartilage, and REJENERA treatment improved Ki67 expression. Furtheremore, immunostaining for Col-2 was also significantly improved in OA rats treated with REJENERA. .  However, IBUPROFEN treatment demonstrated a tendency towards improvement in Ki67, Col-2, BMP-7, and iNOS levels, whilst concurrently exhibiting a reduction in histopathological abnormalities in OA cartilage. This effect was found to be comparable to that observed with REJENERA treatment.

The anti-OA effects of REJENERA are likely to be attributable not only to the pharmacological effects of the ZeyEX polyphenolic components, but also to the effects of its other multi-targeted components. In particular, luteolin has been reported to prevent apoptosis in OA chondrocytes (Goker et al. 2018; Li et al. 2024) and effectively rebalance chondrocyte metabolic activity disrupted by IL-1β (Li et al. 2024). Luteolin has been reported to have the capacity to prevent apoptosis in OA cells (Goker et al. 2018; Li et al. 2024) and to effectively rebalance metabolic activity in these cells that has been disrupted by IL-1β (Li et al. 2024). As with luteolin, quercetin, a senolytic flavonol, also suppresses the development and progression of osteoarthritis by maintaining inflammatory cascade homeostasis in a rat model of osteoarthritis (Wang et al. 2023) and in patients (Yamaura et al. 2023). *S*-allyl-l-cysteine (SAC), a sulfur-containing amino acid found in garlic and one of the main components of REJENERA, has been shown to exhibit a wide range of protective effects, including redox regulatory and anti-inflammatory activities, as well as anti-apoptotic and pro-energetic signalling capacities. As demonstrated in both in vitro (Elmazoglu et al. 2020a, b) and in vivo (Shao et al. 2020) studies, the treatment has been shown to alleviate OA pathology. The sulfhydryl groups in SAC have been demonstrated to suppress NF-κB activation, thereby inhibiting the NF-κB signaling pathway associated with arthritis (Qian et al. 2018). REJENERA also contains palmitoylethanolamide (PEA), a naturally occurring fatty acid amide known for its analgesic and anti-inflammatory effects. Following MIA induction, it has been shown to alleviate cartilage degeneration and knee joint swelling by reducing inflammatory mediators such as NO, PGE2, LTB4, TNF-α, and IL-1β, and suppressing the secretion of cartilage-destroying enzymes such as MMP-1, MMP-3, MMP-9, and MMP-13 (Jung et al. 2021). Boron, an element that is present in REJENERA’s formula, has been shown to have a beneficial effect on bones and joints. As demonstrated in the study by Li et al. (2021), boron plays a significant role in reducing enzymes that trigger the inflammatory response, thus reducing joint pain and stiffness associated with OA. Research has demonstrated that boron has the capacity to stimulate the proliferation of chondrocytes and to promote the formation of cartilage matrix (Bryliński et al. 2025). As posited by de Paz-Lugo et al. (2023), l-proline deficiency is a primary factor in the reduced collagen synthesis observed in OA. l-proline, a pivotal constituent of REJENERA, functions as a preventative agent in the onset of OA by facilitating the restoration of collagen-2 synthesis within cartilage. In addition, exogenous hyaluronic acid has been incorporated into the REJENERA formula due to its reported capacity to inhibit cartilage degeneration and promote regeneration by enhancing hyaluronic acid synthesis in cartilage cells (Migliore and Procopio 2015). Exogenous hyaluronic acid has been reported to play a role in preventing cartilage degeneration and supporting regeneration by increasing the synthesis of hyaluronic acid in cartilage cells (Migliore and Procopio 2015). Hyaluronic acid treatment reduces the production of pro-inflammatory mediators and MMPs (Cicero et al. 2020; Wang et al. 2025).

It is also noteworthy that ZeyEX and REJENERA were administered at the same nominal dose of 300 mg/kg body weight/day, yet one group received 100% ZeyEX while the other received a REJENERA formula containing approximately 15% ZeyEX. The broader activity profile of REJENERA despite the substantially lower ZeyEX content points to a contribution from the additional components of the formula, though the precise nature of any interaction between components—whether additive or otherwise—was not directly determined experimentally in the present study. Nonetheless, REJENERA’s strong inhibitory effect on certain indicators of OA pathology, including the augmentation of NLRP3 expression, suggests that the potential for the components within the formulation to interact synergistically in vivo. On the other hand, the monosodium iodoacetate (MIA) model is proposed as a well-known preclinical tool for studying the pathophysiology of OA and validating “Disease-Modifying Osteoarthritis Drugs”. A substantial body of research posits that intra-articular MIA injection instigates cartilage degeneration, subchondral bone remodelling, and pain by disrupting chondrocyte glycolysis, thus offering a reliable reflection of human OA joint pathology (Sasaki et al. 2024; Song et al. 2025). However, it is imperative to interpret the findings within the limitations imposed by the MIA-induced OA model. MIA has been demonstrated to produce OA-like structural and biochemical changes through the mediation of chemical injury to cartilage cells and rapid disruption of cartilage metabolism. Thus, the therapeutic effects observed with REJENERA and other agents in the MIA model of OA primarily reflect antiinflammatory, antioxidant, anticatabolic, cytoprotective and potentially regenerative activity following chemically induced joint damage.. Although a 12-week period may allow for the assessment of sustained structural and inflammatory changes, the model may not not fully reflect the slow biomechanical and metabolic progression of clinical OA.

## Conclusion

**Fig. 10:**
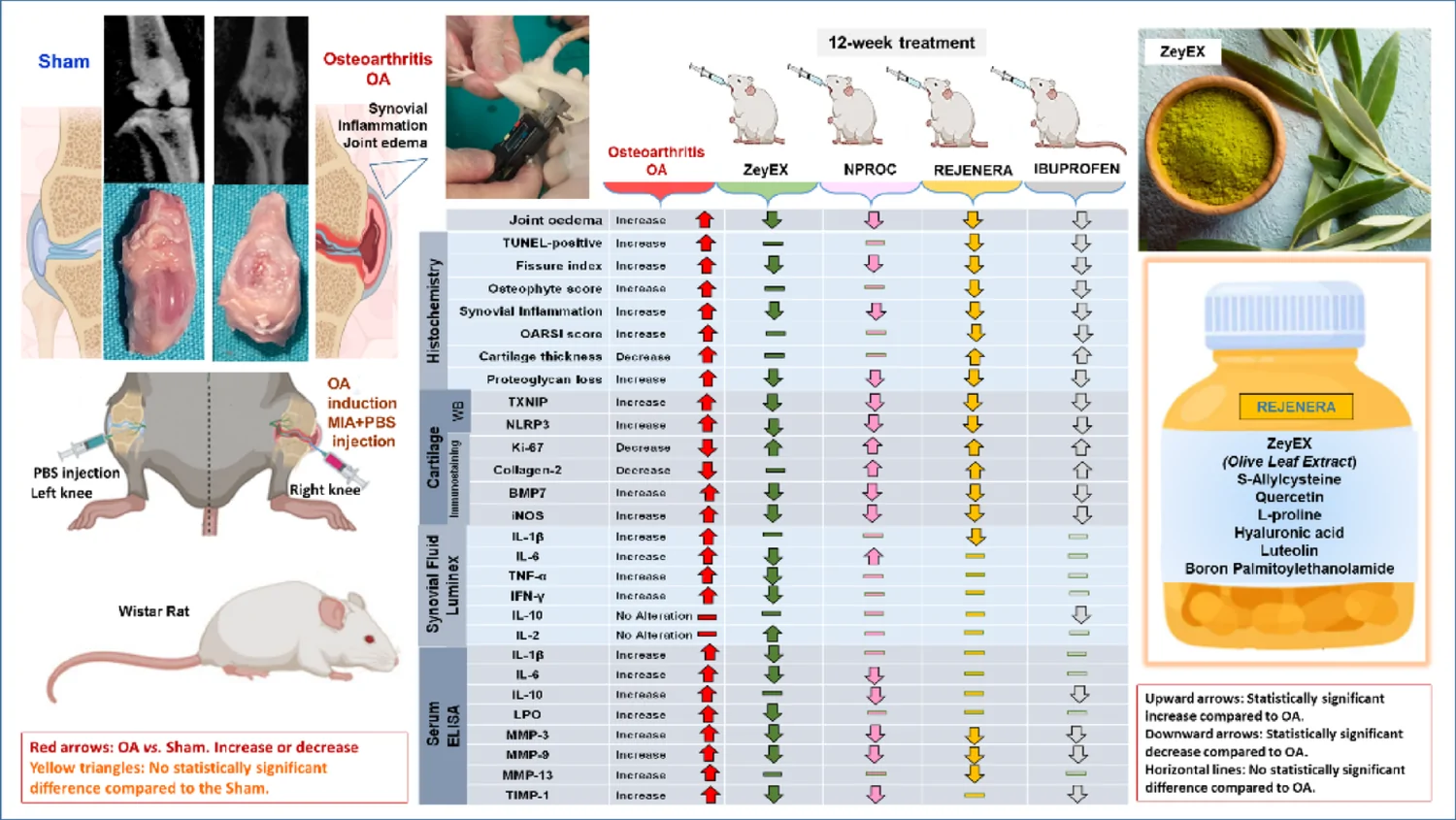
Schematic diagram summarizing the effects of ZeyEX, NPROC, REJENERA, and IBUPROFEN in a rat model of MIA-induced osteoarthritis (OA)

The present study demonstrates that ZeyEX, NPROC, REJENERA, and IBUPROFEN each exert beneficial effects on multiple parameters of OA pathology in the MIA rat model, with distinct profiles across inflammatory, oxidative, and structural markers (The schematic representation is summarized in the graphical abstract) (Fig. 10). ZeyEX demonstrated particular activity in attenuating systemic and synovial cytokine levels and oxidative stress, while REJENERA exhibited a more extensive response across intracellular signaling markers, particularly the TXNIP/NLRP3 axis, MMP-13, iNOS, and cartilage structural proteins, relative to the OA group. These findings underscore the therapeutic potential of multi-component nutraceutical formulations with multiple intracellular targets such as REJENERA in the management of OA, thereby establishing a foundation for subsequent mechanistic and translational investigation.

## Supplementary Information

Below is the link to the electronic supplementary material.


Supplementary Material 1


## Data Availability

No datasets were generated or analysed during the current study.

## Abbreviations

ANOVA: Analysis of variance
BMP-7: Bone morphogenetic protein 7
CMS: Carboxymethyl cellulose
Col-2: Type-2 collagen
DAB: 3,3′ Diaminobenzidine
H&E: Hematoxylin–eosin
iNOS: Inducible nitric oxide synthase
IL-1β: Interleukin-1 beta
IL-2: Interleukin-2
IL-6: Interleukin-6
IL-10: Interleukin-10
IFN-γ: Interferon gamma
Ki-67: Nuclear protein Ki67
LPO: Lipid peroxidation
MIA: Monosodium iodoacetate
MMP-3: Matrix metalloproteinase-3
MMP-9: Matrix metalloproteinase-9
MMP-13: Matrix metalloproteinase-13
NLRP3: NOD-like receptor pyrin domain-containing protein 3
OA: Osteoarthritis
OARSI: Osteoarthritis Research Society International
OS: Oxidative stress
PBS: Phosphate buffer saline
ROS: Reactive oxygen species
SAC: *S*-allylcysteine
TNF-α: Tumor necrosis factor alpha
TdT: Terminal transferase
TIMP-1: Tissue inhibitor of metalloproteinase 1
TUNEL: Terminal deoxynucleotidyl transferase dUTP nick-end labeling
TXNIP: Thioredoxin interacting protein
